# Differential responses to UCP1 ablation in classical brown *versus* beige fat, despite a parallel increase in sympathetic innervation

**DOI:** 10.1016/j.jbc.2024.105760

**Published:** 2024-02-16

**Authors:** Qimuge Naren, Erik Lindsund, Muhammad Hamza Bokhari, Weijun Pang, Natasa Petrovic

**Affiliations:** 1 College of Animal Science and Technology, Northwest A&F University, Yangling, China; 2 Department of Molecular Biosciences, The Wenner-Gren Institute, Stockholm University, Stockholm, Sweden

**Keywords:** UCP1, brown adipose tissue, beige adipose tissue, gene knockout, adipocyte, macrophage, sympathetic nerves, MAO-A, Western blot, immunohistochemistry

## Abstract

In the cold, the absence of the mitochondrial uncoupling protein 1 (UCP1) results in hyper-recruitment of beige fat, but classical brown fat becomes atrophied. Here we examine possible mechanisms underlying this phenomenon. We confirm that in brown fat from UCP1-knockout (UCP1-KO) mice acclimated to the cold, the levels of mitochondrial respiratory chain proteins were diminished; however, in beige fat, the mitochondria seemed to be unaffected. The macrophages that accumulated massively not only in brown fat but also in beige fat of the UCP1-KO mice acclimated to cold did not express tyrosine hydroxylase, the norepinephrine transporter (NET) and monoamine oxidase-A (MAO-A). Consequently, they could not influence the tissues through the synthesis or degradation of norepinephrine. Unexpectedly, in the cold, both brown and beige adipocytes from UCP1-KO mice acquired an ability to express MAO-A. Adipose tissue norepinephrine was exclusively of sympathetic origin, and sympathetic innervation significantly increased in both tissues of UCP1-KO mice. Importantly, the magnitude of sympathetic innervation and the expression levels of genes induced by adrenergic stimulation were much higher in brown fat. Therefore, we conclude that no qualitative differences in innervation or macrophage character could explain the contrasting reactions of brown *versus* beige adipose tissues to UCP1-ablation. Instead, these contrasting responses may be explained by quantitative differences in sympathetic innervation: the beige adipose depot from the UCP1-KO mice responded to cold acclimation in a canonical manner and displayed enhanced recruitment, while the atrophy of brown fat lacking UCP1 may be seen as a consequence of supraphysiological adrenergic stimulation in this tissue.

Understanding of the significance of uncoupling protein 1 (UCP1) for adaptive thermogenesis was advanced by using a mouse model in which UCP1 was ablated (1). This model demonstrated that the ability to replace shivering for nonshivering thermogenesis in the cold is fully dependent on the presence of UCP1 (2); no other adrenergically induced thermogenic mechanism was recruited in the absence of UCP1. However, under conditions that demand *intense* thermoregulatory thermogenesis, brown fat deficient in UCP1 exhibits features not obviously directly related to the absence of the uncoupling function of UCP1. This includes significantly altered mitochondria (3, 4, 5), increased expression and secretion of fibroblast growth factor 21 (4, 6), and infiltration with pro-inflammatory immune cells (3, 7). In contrast, beige (originally referred to as brite (8)) adipose tissue from UCP1-knockout (UCP1-KO) mice also undergoes profound molecular and morphological remodeling in response to cold exposure, but, importantly, toward an apparently more recruited phenotype, characterized by enhanced multilocularity and an increased expression of genes related to thermogenesis and lipolysis (4, 9, 10, 11). This enhanced recruitment of beige fat from the UCP1-KO mice acclimated to cold has recurringly been formulated as a compensatory response leading to the recruitment of thermogenic mechanisms independent of UCP1.

Our goal here was to uncover the mechanisms underlying the differential responses to the cold of classical brown *versus* beige fat from UCP1-KO mice. We confirmed that, in the cold, the ablation of UCP1 resulted in hyper-recruitment of beige fat and also that the classical brown fat lacking UCP1 became atrophied. We then examined whether tissue-selective macrophage accumulation or a different sympathetic innervation could explain these phenomena. We particularly examined the nature of macrophages with respect to their competence to synthesize or catabolize norepinephrine.

## Results

The aim of the present study was to investigate whether the differential cold-induced response to UCP1 ablation in brown *versus* beige fat could be explained by a tissue-selective macrophage accumulation or by a different sympathetic innervation in these tissues. To achieve this, wild-type and UCP1-KO mice were acclimated to thermoneutrality (30 °C) and to two subthermoneutral temperatures: 18 °C (mild cold, close to standard animal house conditions) and 4 °C (conventional cold). Interscapular brown adipose tissue (IBAT), and inguinal white adipose tissue (ingWAT) were selected as representative depots. IBAT is the classical brown fat depot that shows the highest UCP1 expression (12, 13) and is a major brown adipose tissue depot in mice. ingWAT is both the largest beige adipose depot and the one that shows the strongest browning capacity (12, 13).

### Biochemical and morphological characteristics of IBAT and ingWAT in wild-type and UCP1-KO mice

**Figure 1:**
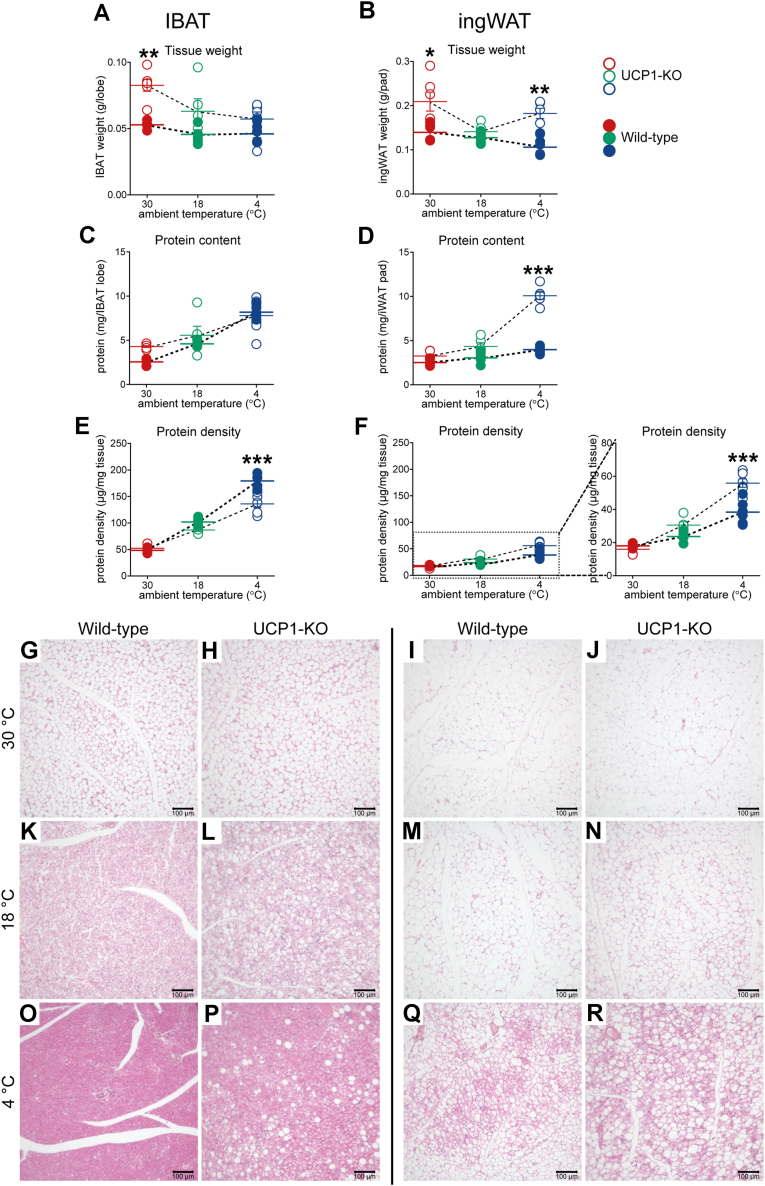
**The effect of UCP1 ablation on biochemical and morphological parameters of IBAT and ingWAT.** Wild-type (filled symbols) (n = 4–6) and UCP1-KO mice (open symbols) (n = 5–6) were acclimated to the indicated temperatures (as detailed in Experimental procedures). *A*, IBAT wet weight, (*B*) ingWAT wet weight, (*C*) IBAT protein content, (*D*) ingWAT protein content, (*E*) IBAT protein density and (*F*) ingWAT protein density. Each symbol represents one mouse sample. Where not visible, the error bars are smaller than the symbols. Values are means ± SEM. Data were analyzed using two-way ANOVA. (Full ANOVA statistics are given in Table S1). ∗Significant difference between wild-type and UCP1-KO mice for each tissue and temperature using the two-way ANOVA followed by Tukey’s multiple comparison test. ∗*p* < 0.05, ∗∗*p* < 0.01, ∗∗∗*p* < 0.001. *G*–*R*, Histological appearance of IBAT (*G*, *H*, *K*, *L*, *O* and *P*) and ingWAT (*I*, *J*, *M*, *N*, *Q* and *R*) from the animals in (*A*–*F*). The tissues were analyzed using hematoxylin and eosin staining, and representative images are presented. Scale bar 100 μm. UCP1-KO, UCP1-knockout.

Biochemical and morphological characteristics of IBAT and ingWAT from wild-type and UCP1-KO mice acclimated to three different temperatures are presented in Figure 1. In wild-type mice (filled symbols), the weights of both IBAT and ingWAT steadily decreased with acclimation to low environmental temperatures (Fig. 1, *A* and *B*) (detailed statistical analysis applying to all Figures is presented in Table S1). Total protein content in each of the tissues increased with acclimation to 18 °C and further increased with acclimation to 4 °C (Fig. 1, *C* and *D*, filled symbols). This increase in total protein content was accompanied by an increased protein density in the tissues (Fig. 1, *E* and *F*, filled symbols) (in adipose tissue, protein density and adiposity are inversely related). Together, these findings suggest cold-induced thermogenic recruitment in both tissues of wild-type mice.

In UCP1-KO mice (open symbols), general IBAT parameters were relatively similar to those in wild-type mice. However, at 4 °C, the protein density in IBAT from UCP1-KO mice was significantly lower than in the tissue from wild-type mice (Fig. 1*E*, blue symbols), suggesting increased adiposity of brown fat deficient in UCP1.

ingWAT parameters in wild-type and UCP1-KO mice displayed larger differences with acclimation to low environmental temperatures (Fig. 1, *B*, *D* and *F*). At 4 °C, ingWAT wet weight in UCP1-KO mice was significantly larger (Fig. 1*B*, blue symbols), and this was accompanied by a marked increase in tissue protein content (Fig. 1*D*, blue symbols) and protein density (Fig. 1*F*, blue symbols). This higher wet weight was therefore not a consequence of an increased lipid content in the tissue but was rather an indication of a greater tissue recruitment.

To verify the consequences of UCP1 ablation on IBAT *versus* ingWAT morphology, the tissues were stained with hematoxylin and eosin. Examples are shown in Figure 1, *G*–*R* and are here only qualitatively described as the morphology is principally in agreement with earlier observations as detailed. At thermoneutrality, the majority of brown adipocytes show lipids accumulated in a single large unilocular lipid droplet, as seen earlier (*e.g.* (14, 15, 16)); no difference was observed between wild-type and UCP1-KO mice (Fig. 1, *G* and *H*). The brown adipocytes in wild-type mice acclimated to subthermoneutral temperatures had typical multilocular lipid droplet morphology (Fig. 1, *K* and *O*). The majority of brown adipocytes in UCP1-KO mice acclimated to 18 °C and to 4 °C were also multilocular but a number of adipocytes contained only a single large lipid droplet (as shown in (4)) (Fig. 1, *L* and *P*). The ingWAT of mice acclimated to thermoneutrality was composed of only unilocular adipocytes (in agreement with *e.g.* (14, 15, 16)); no difference was observed between wild-type and UCP1-KO mice (Fig. 1, *I* and *J*). In wild-type mice acclimated to 18 °C, paucilocular adipocytes emerged only sporadically (Fig. 1*M*). Paucilocular/multilocular adipocytes in UCP1-KO mice appeared to be more prevalent, appearing in a few islands surrounded by unilocular adipocytes (Fig. 1*N*). In mice acclimated to stronger cold (4 °C), multilocular adipocytes became even more conspicuous, forming numerous islands surrounded by unilocular adipocytes. Notably, the islands in ingWAT from UCP1-KO mice (Fig. 1*R*) appeared to be larger compared to those in ingWAT from wild-type mice (Fig. 1*Q*) (in agreement with *e.g.* (4, 6, 9)).

These observations at both a histological and a biochemical level essentially confirm previously reported data, such as increased adiposity in brown fat and significantly higher protein content as well as lower adiposity in beige fat from the UCP1-KO mice acclimated to the cold. They thus indicate an apparently regular recruitment of classical brown fat and an enhanced recruitment of brite/beige fat in UCP1-KO mice exposed to cold. An important question relating to this is the nature of proteins that underlie these recruitment processes.

### In the cold, the ablation of UCP1 triggers macrophage accumulation in both brown and beige adipose depots

Earlier studies demonstrated that exposure of UCP1-KO mice to subthermoneutral temperatures promotes brown-fat inflammation characterized by increased levels of proteins involved in host defense, macrophage infiltration, and formation of crown-like structures (3, 7). Macrophage recruitment to ingWAT of UCP1-KO mice has not been studied previously but could provide important insights into the mechanisms underlying different responses to cold in IBAT *versus* ingWAT from these mice. We therefore also examined macrophage recruitment to the beige fat.

**Figure 2:**
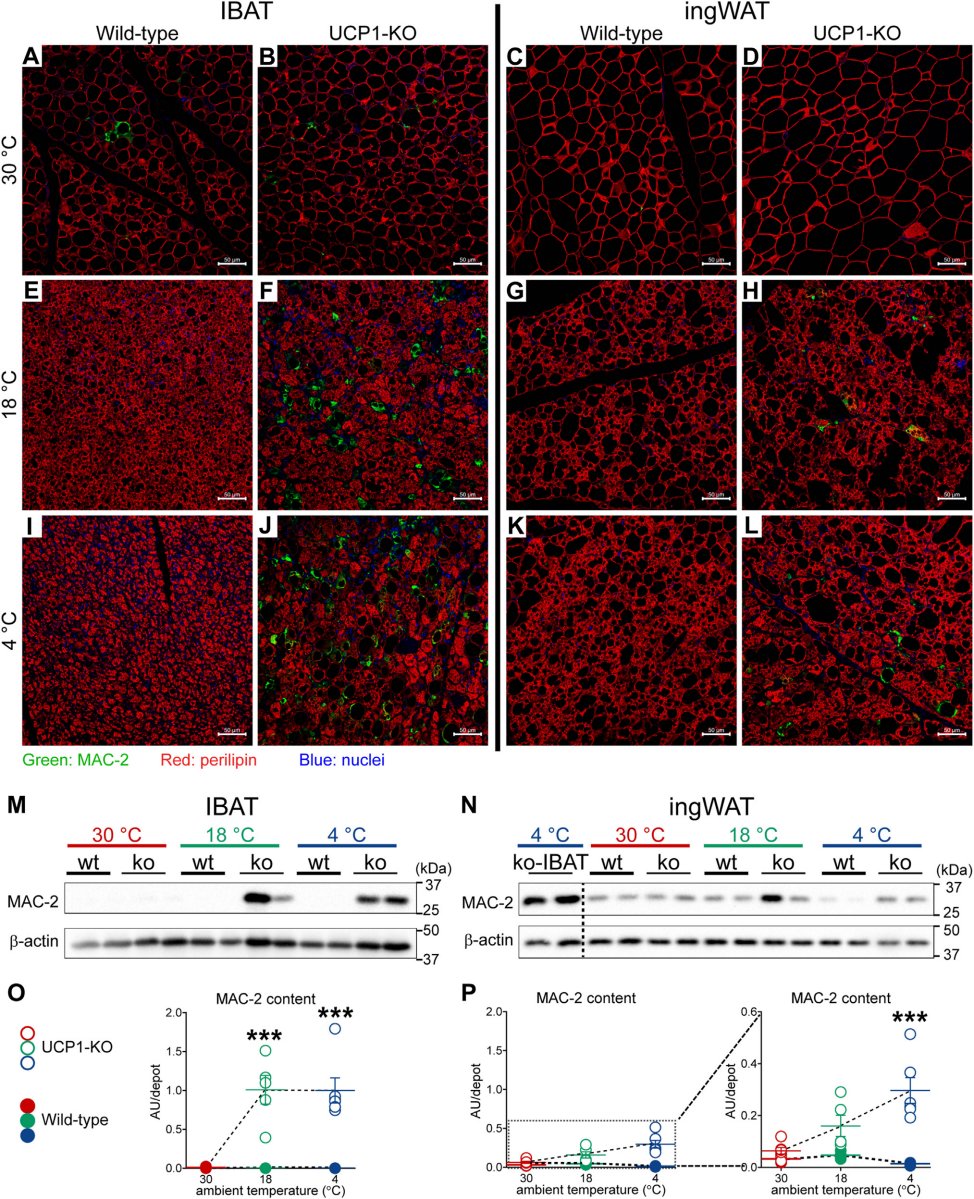
**A notable accumulation of macrophages in IBAT and ingWAT of UCP1-KO mice housed below thermoneutrality.***A*–*L*, representative confocal images of IBAT (*A*, *B*, *E*, *F*, *I* and *J*) and ingWAT (*C*, *D*, *G*, *H*, *K* and *L*) from wild-type and UCP1-KO mice acclimated to 30 °C, 18 °C and 4 °C. The tissues were stained for MAC-2 (*green*), perilipin (*red*) and nuclei (*blue*). Scale bar 50 μm. *M* and *N*, representative western blots of MAC-2 in IBAT (*M*) and ingWAT (*N*) from animals acclimated to the indicated temperatures. *O* and *P*, MAC-2 content in IBAT (*O*) and ingWAT (*P*) in the indicated samples. The mean value in IBAT of UCP1-KO mice acclimated to 4 °C was set to 1.0, and the levels in all other samples were expressed relative to this value. Each symbol represents a sample from one mouse. Values are means ± SEM. Where not visible, the error bars are smaller than the symbols. Full ANOVA statistics are given in Table S1. ∗Significant difference between wild-type and UCP1-KO mice for each tissue using two-way ANOVA followed by Tukey’s multiple comparison test. ∗∗∗*p* < 0.001. To facilitate comparisons of MAC-2 levels between IBAT and ingWAT, the respective graphs were drawn with equal y-axis range. In the right panel of P, the graph was redrawn with optimal y-axis range. UCP1-KO, UCP1-knockout.

The morphology, frequency, and distribution of macrophages in the tissues were first examined qualitatively using immunohistochemistry (Fig. 2, *A*–*L*). Macrophages were visualized with an anti-MAC-2 antibody (green); MAC-2, also known as galectin-3 or LGALS3, is a lectin that mediates macrophage phagocytic and inflammatory responses (17, 18). The adipocytes were visualized by staining the lipid-droplet protein perilipin (red). At thermoneutrality, macrophages in brown fat were sporadically found and organized into crown-like structures (Fig. 2, *A* and *B*); no difference was observed between the tissues from wild-type (Fig. 2*A*) and UCP1-KO mice (Fig. 2*B*). In the brown fat from wild-type mice acclimated to 18 °C and to 4 °C, macrophages could not be visually observed (Fig. 2, *E* and *I*). This result is in full agreement with our earlier report demonstrating the presence of macrophages in brown fat from wild-type mice acclimated to thermoneutral temperature and no increase or even downregulation in their abundance upon exposure to cold (19, 20). However, importantly, in clear contrast to the case in the wild-type mice, in the UCP1-KO mice, acclimation to subthermoneutral temperatures resulted in a massive macrophage accumulation into brown fat (Fig. 2, *F* and *J*) (in agreement with ((3, 7))). The majority of these macrophages were aggregated into crown-like structures (Fig. 2, *F* and *J*), while a few macrophages were scattered within the tissue as solitary macrophages (for details see enlarged version of Fig. 2*J* (Fig. S1)).

In ingWAT, in contrast to the case in IBAT, no macrophages could be seen in mice acclimated to thermoneutrality (Fig. 2, *C* and *D*). In mice acclimated to subthermoneutral temperatures, macrophages were found only in ingWAT from UCP1-KO mice (Fig. 2, *H* and *L*) (for details see enlarged version of Fig. 2*L* (Fig. S2)). Thus, similar to the case in brown fat, under cold conditions, the ablation of UCP1 promotes macrophage accumulation in beige adipose depots; the effects seen in ingWAT were qualitatively similar but less robust compared to those in IBAT.

To estimate macrophage content in the tissues, we quantified MAC-2 protein using immunoblot analysis (Fig. 2, *M* and *N*). As seen in Figure 2*M*, in the IBAT, MAC-2 protein could essentially be detected only in tissues from UCP1-KO mice exposed to subthermoneutral temperatures. Immunoblot analysis failed to detect the MAC-2 protein in the IBAT from the mice acclimated to thermoneutrality, likely due to an immense difference in expression levels. In support of this, the analysis of only the samples from wild-type mice (Fig. S3) was fully consistent with our earlier report in which MAC-2 protein had been readily detected in similar samples (19). The total MAC-2 protein in the IBAT (Fig. 2*O*) was calculated by multiplying the protein levels of MAC-2 expressed per mg tissue protein (Fig. S4) with the total protein content in the tissue (Fig. 1*C*) and thus was found to display nearly infinite-fold induction upon acclimation of UCP1-KO mice to subthermoneutral temperatures. This observation was further supported by the analysis of a number of macrophage marker genes; the expression levels of all analyzed marker genes (general and M1 and M2 macrophage types) were highest in samples from UCP1-KO mice acclimated to 4 °C (Fig. S5).

Immunoblot analysis of MAC-2 in ingWAT revealed a discrepancy with the immunostaining results. Despite the absence of visually observable macrophages in the tissues from wild-type mice acclimated to subthermoneutral temperatures (Fig. 2, *G* and *K*), the MAC-2 protein in these tissues was clearly detectable by immunoblotting (Fig. 2*N*). This discrepancy may be attributed to the macrophages residing in the small lymph nodes within ingWAT, which may not have been fully removed during the dissection. In support of this notion is the significantly higher expression (approximately 5–10-fold) of several general macrophage marker genes in ingWAT compared to IBAT in cold-acclimated wild-type mice (Fig. S6), both of which contain negligible amounts of macrophages (Fig. 2, *I* and *K*). Due to the intricate connection between the adipose and lymphatic compartments in ingWAT, the levels of MAC-2 protein in ingWAT can only be approximately estimated. Despite this limitation, the results of immunoblot analysis of MAC-2 in ingWAT (Fig. 2, *N* and *P*) were qualitatively similar to those obtained in IBAT (Fig. 2, *M* and *O*), but the differences between wild-type and UCP1-KO mice were less drastic.

We thus confirm here that in the cold, the ablation of UCP1 triggers macrophage accumulation into brown adipose tissue. Importantly, we demonstrate that this is also the case in beige adipose tissue and thus, the phenomenon as such is unlikely to explain the differential response to UCP1 ablation in brown *versus* beige fat upon exposure to cold, where brown fat undergoes atrophy, while beige fat becomes hyper-recruited. This finding thus raises the question as to whether the macrophages occurring in brown and beige fat differ in their competence to affect adrenergic signaling in the tissue.

### No norepinephrine-producing capacity of adipose tissue macrophages

Adipose tissue macrophages (alternatively activated), due to their suggested ability to synthesize and release norepinephrine, have earlier been proposed to directly stimulate adaptive thermogenesis in brown and beige adipose tissues (21). However, in an earlier study (19), we demonstrated that the macrophages accumulating in brown fat during prolonged exposure to thermoneutrality did not express tyrosine hydroxylase, the enzyme catalyzing the first and rate-limiting step in catecholamine biosynthesis (22). This agrees with the observation that the macrophages occurring in brown fat of mice acclimated to subthermoneutral temperatures (21 °C and 4 °C) do not synthesize catecholamines or contribute to adipose tissue adaptive thermogenesis (23). However, importantly, brown fat from those mice contained macrophages only at low densities. In contrast to that study, the brown and beige fat analyzed here from the UCP1-KO mice acclimated to 18 °C or to 4 °C were heavily infiltrated by macrophages. We examined whether these (or some of these) abundantly present macrophages possessed the capacity to produce catecholamines, potentially in a tissue-specific manner.

**Figure 3:**
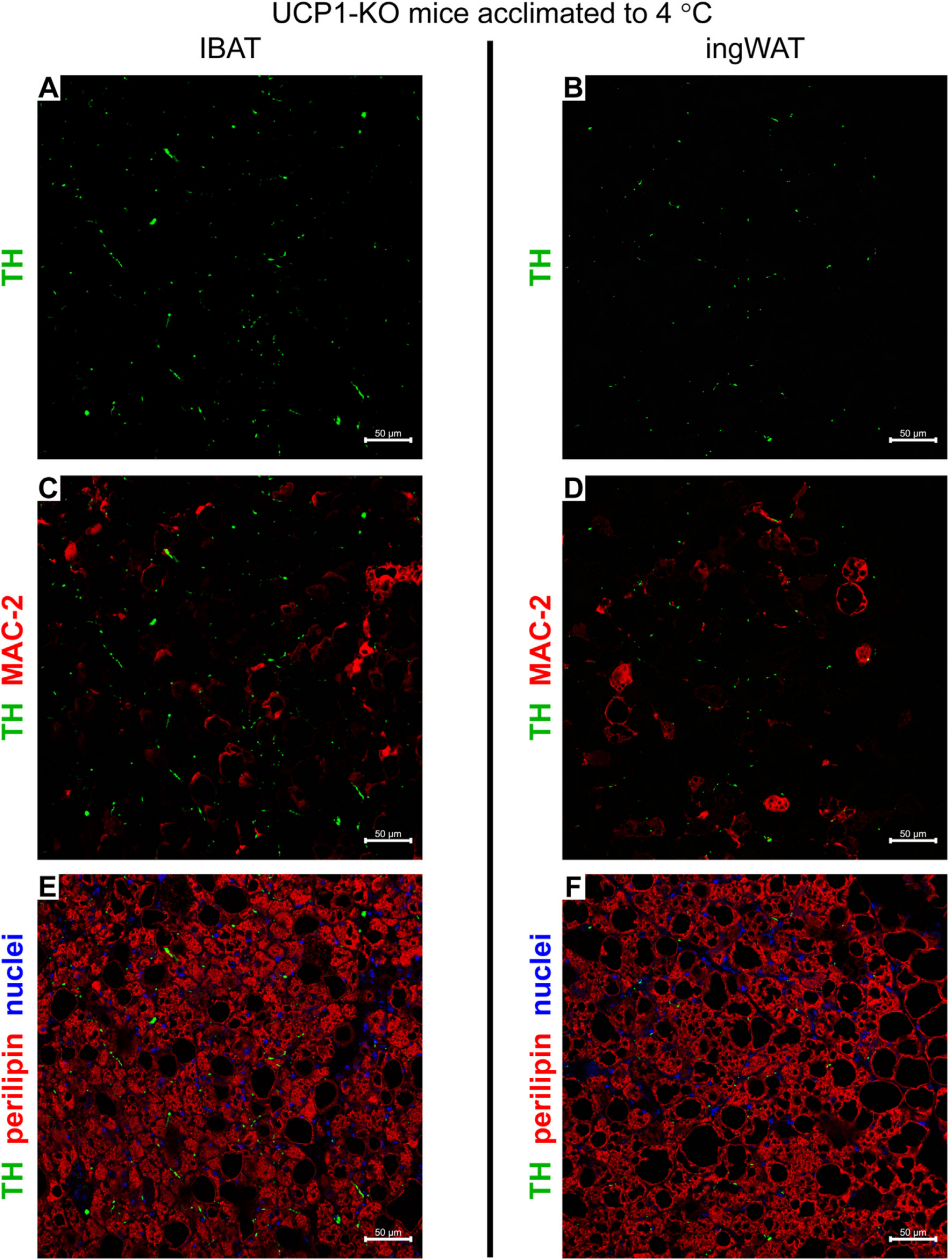
**Macrophages in IBAT and ingWAT of UCP1-KO mice housed below thermoneutrality do not express tyrosine hydroxylase.***A*–*F*, representative confocal images of IBAT (*A*, *C* and *E*) and ingWAT (*B*, *D* and *F*) from UCP1-KO mice acclimated to 4 °C. The tissues were stained for tyrosine hydroxylase (TH) (*green*), MAC-2 (*red*) (*C* and *D*), perilipin (red pseudocolor) (*E* and *F*) and nuclei (*blue*). Scale bar 50 μm. UCP1-KO, UCP1-knockout.

If macrophages in IBAT and ingWAT from UCP1-KO mice acclimated to subthermoneutral temperatures should be able to synthesize norepinephrine, this would require that they were endowed with tyrosine hydroxylase. We therefore investigated the presence and localization of tyrosine hydroxylase protein *in situ* using immunohistochemistry. Tyrosine hydroxylase protein was readily detected in both IBAT and ingWAT and appeared as dotted or filamentous staining (green) (Fig. 3, *A* and *B*). Importantly, as seen in Figure 3, *C* and *D*, no tyrosine hydroxylase immunoreactivity was detected within macrophages (red) (for details see enlarged version of Fig. 3*C* (Fig. S7)). Visualization of adipocytes with an anti-perilipin antibody (now stained red) (Fig. 3, *E* and *F*) demonstrated that these dotted and filamentous tyrosine hydroxylase-positive structures were closely associated with adipocytes but were never found *within* adipocytes (nor in any other cell type). Therefore, the tyrosine-hydroxylase-positive filamentous structures most likely correspond to sympathetic fibers, whereas the dotted structures represent the boutons-en-passant, the contact sites between the nerves and the adipocytes.

Taken together, these observations expand previous data (obtained in different mouse models) that the macrophages infiltrating brown and beige adipose tissues do not express tyrosine hydroxylase and are therefore unable to synthesize catecholamines (19, 23)). Consequently, the differences in response to cold in IBAT *versus* ingWAT from UCP1-KO mice cannot be explained by a tissue-specific macrophage population competent in norepinephrine synthesis. Thus, the norepinephrine found in brown and beige adipose tissues must be of exclusively neuronal origin.

### No evidence for norepinephrine-catabolizing capacity in a majority of adipose tissue macrophages

From the above data, it was concluded that the vast number of macrophages accumulated within brown and beige fat of UCP1-KO mice acclimated to cold could not affect the tissues through the synthesis of norepinephrine. However, two other studies have provided a novel aspect of macrophage function with respect to the regulation of adaptive thermogenesis (24, 25). Those studies suggested the existence of a subpopulation of macrophages, found around sympathetic nerves of subcutaneous (sympathetic neuron-associated macrophages (SAMs)) (24) or visceral (nerve-associated macrophages (NAMs)) (25) adipose tissue, that displayed a high expression of the norepinephrine-degrading enzyme monoamine oxidase-A (MAO-A). The SAMs also displayed a high expression of the neuronal norepinephrine transporter (NET) (official gene name: solute carrier family six member 2 (SLC6A2)). Therefore, these specialized macrophages have been ascribed a norepinephrine-lowering and thus thermogenesis-regulatory function.

**Figure 4:**
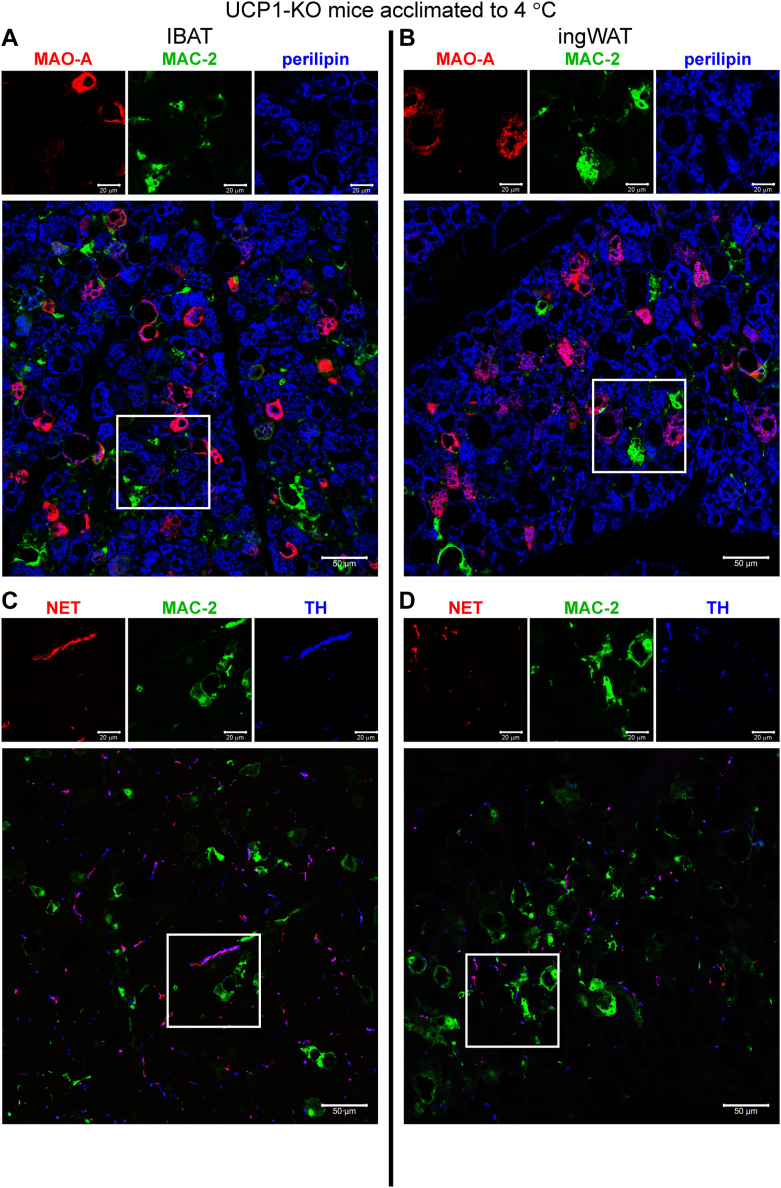
**Macrophages in IBAT and ingWAT of UCP1-KO mice housed below thermoneutrality are not equipped with the enzymes necessary for norepinephrine catabolism.***A*–*D*, representative confocal images of IBAT (*A* and *C*) and ingWAT (*B* and *D*) from UCP1-KO mice acclimated to 4 °C. In (*A* and *B*), the tissues were stained for monoamine oxidase-A (MAO-A) (*red*), MAC-2 (*green*) and perilipin (*blue* pseudocolor). In (*C* and *D*), the tissues were stained for norepinephrine transporter (NET) (*red*), MAC-2 (*green*) and TH (blue pseudocolor). Scale bar 50 μm. Individual antibody staining is shown in the top-inset panels (scale bar 20 μm). Images were acquired with settings in each case allowing the maximum signal detection below the saturation limits of the detectors and are therefore not directly quantitatively comparable. Note that the MAO-A staining (*red* in *A* and *B*) overlapped only with perilipin staining (*blue*). Similarly, the NET staining (*red* in *C* and *D*) overlapped only with TH staining (*blue*). UCP1-KO, UCP1-knockout.

To reveal whether these specialized macrophages displayed tissue-specific distribution and whether they, by being competent in taking up and catabolizing norepinephrine, could be part of the mechanism underlying the differences between UCP1-deficient IBAT and ingWAT in response to cold, we examined the presence and localization of MAO-A and NET *in situ* using immunohistochemistry (Fig. 4, *A*–*D*). MAO-A (red) was readily detected in a large number of cells of both IBAT and ingWAT (Fig. 4, *A* and *B*). Nearly all cells positive for MAO-A were negative for MAC-2 and positive for perilipin (for details see enlarged Fig. 4*A* (Fig. S8)); only a few cells positive for MAO-A displayed ambiguous staining patterns (they also exhibited positive but faint immunoreactivity for both MAC-2 and perilipin) (Fig. S8, white arrows). Importantly, the NET immunoreactivity was exclusively localized to the sympathetic nerves—it was found within filamentous and punctate structures that were also positive for tyrosine-hydroxylase and was never found within macrophages (Fig. 4, *C* and *D*). Thus, no macrophages endowed with NET and MAO-A could be detected either in brown or in beige adipose tissue from the UCP1-KO mice acclimated to cold. Consequently, the differences in response to cold between IBAT and ingWAT from UCP1-KO mice acclimated to cold cannot be explained by a tissue-specific population of macrophages competent in metabolizing norepinephrine. However, we should emphasize that our inability to identify NET- and MAO-A-positive macrophages does not necessarily totally exclude the existence of SAMs/NAMs in these macrophage-rich tissues, but it does suggest that the bulk of the macrophages were not competent to affect the tissues through lowering norepinephrine availability.

### Cold exposure specifically induces the expression of MAO-A exclusively in UCP1-KO brown and beige adipocytes

**Figure 5:**
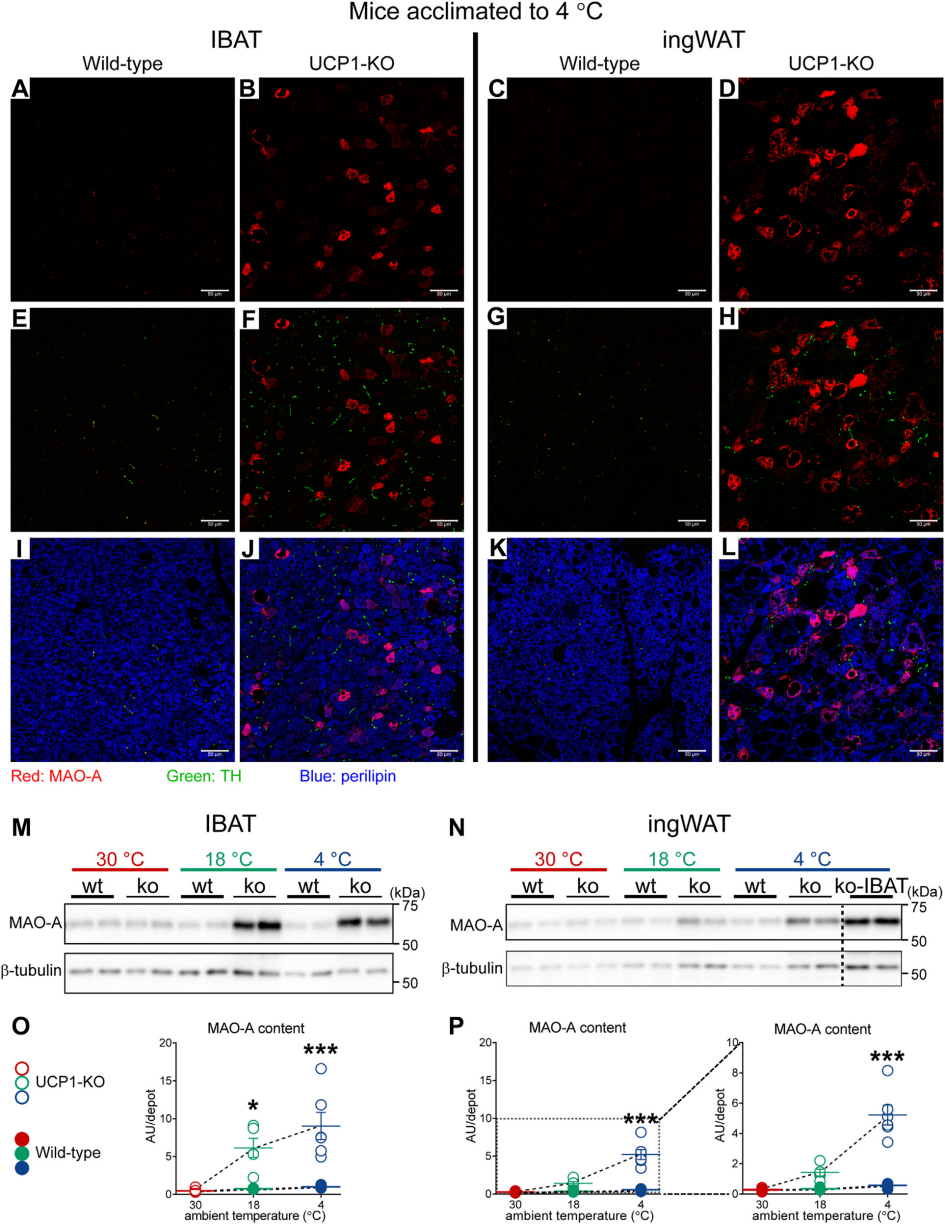
**A strong induction and unexpected adipocyte localization of MAO-A in IBAT and ingWAT of UCP1-KO mice housed below thermoneutrality.***A*–*L*, Representative confocal images of IBAT (*A*, *B*, *E*, *F*, *I* and *J*) and ingWAT (*C*, *D*, *G*, *H*, *K* and *L*) from wild-type and UCP1-KO mice acclimated to 4 °C. The tissues were stained for MAO-A (*red*), TH (*green*) and perilipin (*blue* pseudocolor). Scale bar 50 μm. Images were acquired with settings in each case allowing the maximum signal detection below the saturation limits of the detectors and are therefore not directly quantitatively comparable. *M* and *N*, representative western blots of MAO-A and β-tubulin (cannot be used as a loading control due to the plasticity of the tissues) in IBAT (*M*) and ingWAT (*N*) from animals acclimated to the indicated temperatures. *O*, IBAT MAO-A content and (*P*) ingWAT MAO-A content in the indicated samples. The mean value in IBAT of wild-type mice acclimated to 4 °C was set to 1.0, and the levels in all other samples were expressed relative to this value. Each symbol represents a sample from one mouse. Values are means ± SEM. Where not visible, the error bars are smaller than the symbols. ∗Significant difference between wild-type and UCP1-KO mice for each tissue using two-way ANOVA followed by Tukey’s multiple comparison test. ∗*p* < 0.05, ∗∗∗*p* < 0.001. To facilitate comparisons of MAO-A levels between IBAT and ingWAT, the respective graphs were drawn with equal y-axis range. In the right panel of P, the graph was redrawn with optimal y-axis range. UCP1-KO, UCP1-knockout.

As shown earlier, in brown and beige adipose tissues from the UCP1-KO cold-acclimated mice, MAO-A protein was detectable in the population of cells expressing perilipin, which thus corresponds to the adipocytes. However, in light of a recent report showing that in mice MAO-A was not expressed in adipocytes (26), this observation is unexpected and surprising. Importantly, MAO-A activity is readily measured in rat brown adipose tissue (*e.g.* (27)), but MAO-A is primarily considered an intraneuronal enzyme (28). To clarify whether MAO-A is innately expressed in brown and beige adipocytes, or its expression is selectively induced by UCP1 ablation, we analyzed the distribution of MAO-A immunoreactivity in tissues from both wild-type and UCP1-KO mice acclimated to cold (Fig. 5, *A*–*L*). In the same sections, sympathetic nerves were visualized with an anti-tyrosine hydroxylase antibody (green) and adipocytes with an anti-perilipin antibody (blue).

Tissue sections from wild-type mice displayed very scarce MAO-A immunoreactivity, with dotted or filamentous appearance (red) (Fig. 5, *A* and *C*), which entirely overlapped with tyrosine hydroxylase staining (Fig. 5, *E* and *G*). Thus, in brown and beige fat from wild-type mice, MAO-A displayed only neuronal (sympathetic) localization. In the tissues from UCP1-KO mice, a large number of cells exhibited strong MAO-A immunoreactivity (Fig. 5, *B* and *D*) (see also Fig. 4, *A* and *B*). However, MAO-A was not confined to adipocytes (Fig. 5, *J* and *L*), but was also localized to sympathetic nerves (Fig. 5, *F* and *H*). The staining intensity in nerves was weaker and could only be clearly visualized in the overexposed images (see enlarged and overexposed Fig. 5*B* (Fig. S9)).

Thus, we confirm that mouse adipocytes do not innately express MAO-A (in agreement with (26)), but upon ablation of UCP1, they become competent to express this norepinephrine-degrading enzyme. Importantly, although the brown and beige adipocytes display different anatomical localization and different molecular signatures (8), and are also of different embryological origin (29, 30), they do not differ in their ability to acquire expression of MAO-A in response to the ablation of UCP1 and the exposure to cold.

To quantify MAO-A in IBAT and ingWAT from wild-type and UCP1-KO mice, we quantified the MAO-A protein levels using immunoblot analysis (Fig. 5, *M* and *N*). In the mice acclimated to thermoneutrality, MAO-A protein levels (expressed per mg tissue protein) did not differ between the two genotypes in either of the tissues (Figs. 5, *M* and *N* and S10). However, under subthermoneutral conditions (18 °C and 4 °C), MAO-A levels were notably increased in both IBAT and ingWAT from UCP1-KO mice (Figs. 5, *M* and *N* and S10). MAO-A protein content in the total tissues was calculated by multiplying MAO-A protein levels expressed per mg tissue protein (Fig. S10) with the total protein content in the tissue (Fig. 1, *C* and *D*). As shown in Figure 5, *O* and *P* and in Table S1, the MAO-A content was only increased in the tissues from the UCP1-KO mice acclimated to 18 °C and 4 °C and was there approximately 10-fold higher than in the tissues from wild-type mice (in agreement with immunostaining data).

Therefore, it may be said that in cold, the brown and beige adipocytes lacking UCP1 acquired what appears to be a distinct neurogenic feature—the ability to express MAO-A—and thus also the potential to catabolize norepinephrine. As MAO-A is a mitochondrial outer membrane-bound enzyme that, by catalyzing deamination of catecholamines generates hydrogen peroxide, it may play a significant metabolic role not only by lowering norepinephrine availability but also by increasing oxidative stress in the tissues (see Discussion).

### In the cold, the ablation of UCP1 results in notably enhanced sympathetic innervation in both brown and beige adipose depots

**Figure 6:**
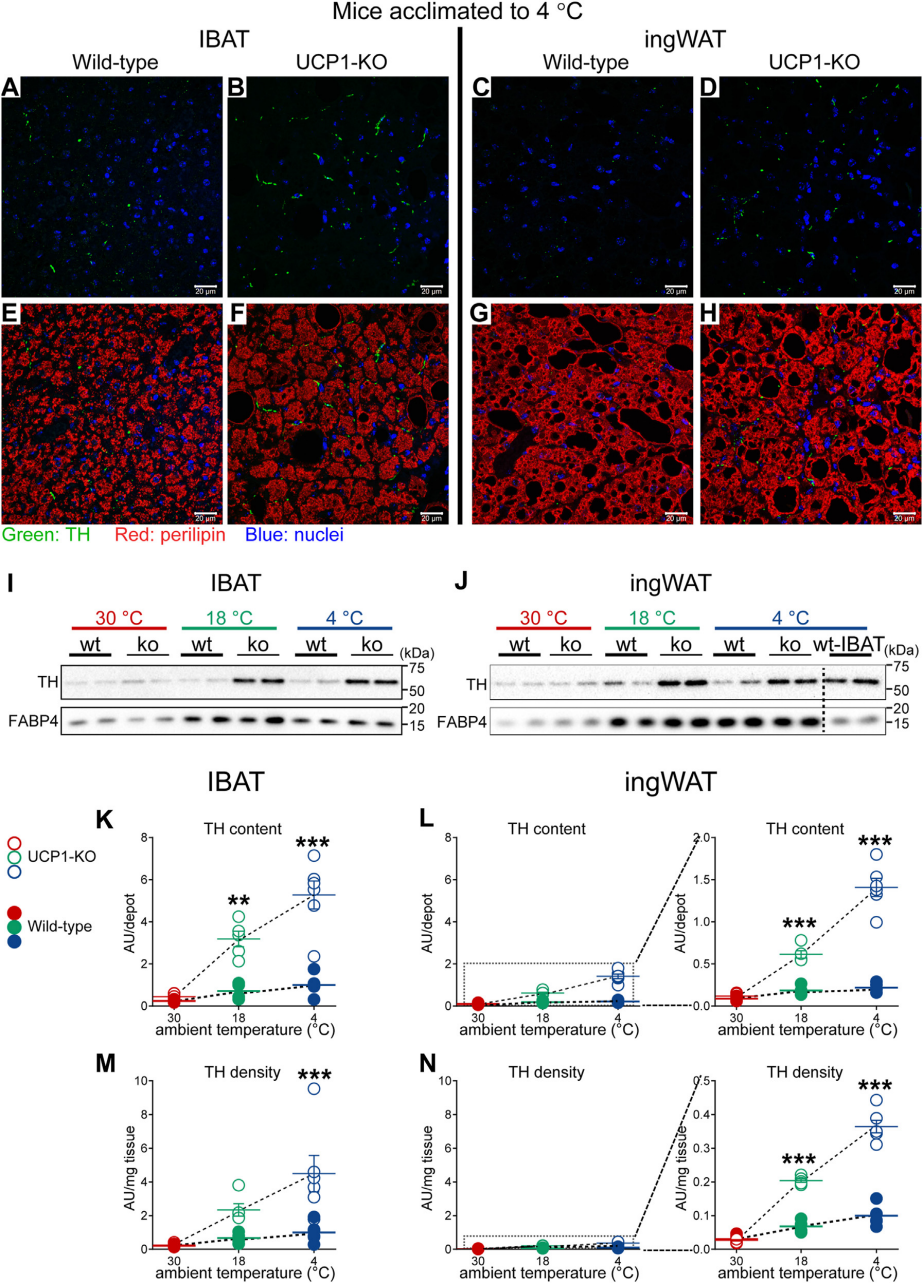
**Notably enhanced sympathetic innervation in IBAT and ingWAT of UCP1-KO mice housed below thermoneutrality.***A*–*H*, representative confocal images of IBAT (*A*, *B*, *E* and *F*) and ingWAT (*C*, *D*, *G* and *H*) from wild-type and UCP1-KO mice acclimated to 4 °C. The tissues were stained for TH (*green*), perilipin (*red*) and nuclei (*blue*). Scale bar 20 μm. Images were acquired with settings in each case allowing the maximum signal detection below the saturation limits of the detectors and are therefore not directly quantitatively comparable. *I* and *J*, representative western blots of tyrosine hydroxylase (TH) and FABP4 (was not used as a loading control) in IBAT (*I*) and ingWAT (*J*) from animals acclimated to the indicated temperatures. *K*, IBAT TH content, (*L*) ingWAT TH content, (*M*) IBAT TH density, and (*N*) ingWAT TH density in the indicated samples. The value in IBAT of wild-type mice acclimated to 4 °C was set to 1.0, and the levels in all other samples were expressed relative to this value. Each symbol represents a sample from one mouse. Values are means ± SEM. Where not visible, the error bars are smaller than the symbols. ∗Significant difference between wild-type and UCP1-KO mice for each tissue using two-way ANOVA followed by Tukey’s multiple comparison test. ∗∗*p* < 0.01, ∗∗∗*p* < 0.001. To facilitate comparisons of TH levels between IBAT and ingWAT, the respective graphs were drawn with equal y-axis range. In the right panels of *L* and *N*, the graphs were redrawn with optimal y-axis range. UCP1-KO, UCP1-knockout.

The above-observed recruitment of the norepinephrine-catabolizing enzyme MAO-A in the brown and beige adipose tissues from UCP1-KO mice acclimated to subthermoneutral temperatures would seem to indicate an increased norepinephrine-turnover in these tissues. This implies that the norepinephrine-producing pathways should also be increased. As shown earlier (19, 23), and also in this study (see above), within adipose tissues, tyrosine hydroxylase is exclusively localized to the sympathetic nerves. Thus, in these tissues, norepinephrine can only be of sympathetic origin. To explore whether there was an effect of UCP1 ablation on the level of sympathetic innervation in brown *versus* beige adipose tissue, we qualitatively (using immunohistochemistry) evaluated the degree of sympathetic innervation in each of the tissues (Fig. 6, *A*–*H*).

Sympathetic nerves were visualized with an anti-tyrosine hydroxylase antibody (green). For clearer visualization of nerve fibers, in the upper panels, only tyrosine hydroxylase and nuclei (blue) are presented (Fig. 6, *A*–*D*). Counterstaining for perilipin (red) enabled visualization of adipocytes (Fig. 6, *E*–*H*). Even with the limitation that the images cannot be directly quantitatively compared (due to the higher exposure times for ingWAT samples, see Experimental procedures), it is evident that in mice acclimated to 4 °C, the tyrosine hydroxylase staining was much less abundant in ingWAT than in IBAT in both of the genotypes (Fig. 6, *C*, *D*, *G* and *H*). Importantly, the density of TH staining in the tissues from UCP1-KO mice (Fig. 6, *B*, *F*, *D* and *H*) appeared notably greater than in the corresponding tissues from wild-type mice (Fig. 6, *A*, *E*, *C* and *G*) and thus indicated an enhanced sympathetic innervation in these tissues.

The degree of sympathetic innervation was then examined quantitatively by measuring the levels of tyrosine hydroxylase protein in IBAT and ingWAT using immunoblot analysis (Fig. 6, *I*–*N*). In the mice acclimated to thermoneutrality, protein levels of tyrosine hydroxylase in each of the tissues did not differ between the two genotypes (Figs. 6, *I* and *J* and S11). However, under subthermoneutral conditions (18 °C and 4 °C), the levels in both IBAT and ingWAT from UCP1-KO mice were notably increased in comparison to the levels in the corresponding tissues of wild-type mice (Figs. 6, *I* and *J* and S11). To estimate the degree of sympathetic innervation in the entire brown and beige adipose depots, we calculated tyrosine hydroxylase protein content in the whole depots by multiplying the tyrosine hydroxylase protein levels expressed per mg tissue protein (Fig. S11) with the total protein content in the tissue (Fig. 1, *C* and *D*). As shown in Figure 6, *K* and *L* and in Table S1, the tyrosine hydroxylase content in both tissues in each genotype increased with exposure to increased cold stress. Notably, the tyrosine hydroxylase content in the tissues from the UCP1-KO mice was about 6-fold higher than in the corresponding tissues from the wild-type mice; in ingWAT from the UCP1-KO mice acclimated to 4 °C (Fig. 6*L*, empty blue symbols), the tyrosine hydroxylase content reached values similar to those in IBAT from the wild-type mice (Fig. 6*K*, filled blue symbols). The remarkably elevated tyrosine hydroxylase content in tissues from UCP1-KO mice acclimated to subthermoneutral temperatures indicates a substantial increase in norepinephrine-producing capacity.

To further characterize the consequence of UCP1 ablation on sympathetic innervation in brown *versus* beige adipose tissues, we estimated the density of sympathetic innervation in each tissue type. The relative content of tyrosine hydroxylase protein per unit tissue weight (tyrosine hydroxylase density) was much lower in ingWAT than in IBAT regardless of the genotype (Fig. 6, *M* and *N*). Importantly, tyrosine hydroxylase protein density in both tissues gradually increased with exposure to decreased environmental temperatures and was also much higher in the tissues from UCP1-KO mice, which agrees with the qualitative observations presented in Figure 6, *A*–*H*.

Thus, in the cold, the ablation of UCP1 evoked hyper-innervation of both brown and beige adipose tissues although the levels were much higher in brown fat.

### The ablation of UCP1 markedly augments the expression of adrenergically regulated thermogenic marker genes

The enhanced sympathetic innervation may not necessarily be accompanied by an increased sympathetic activity. We therefore sought to estimate the magnitude of sympathetic activity in these tissues. We employed an indirect approach by determining expression levels of adipocyte-specific/selective thermogenic marker genes known to be induced by adrenergic signaling (as discussed in (31)).

**Figure 7:**
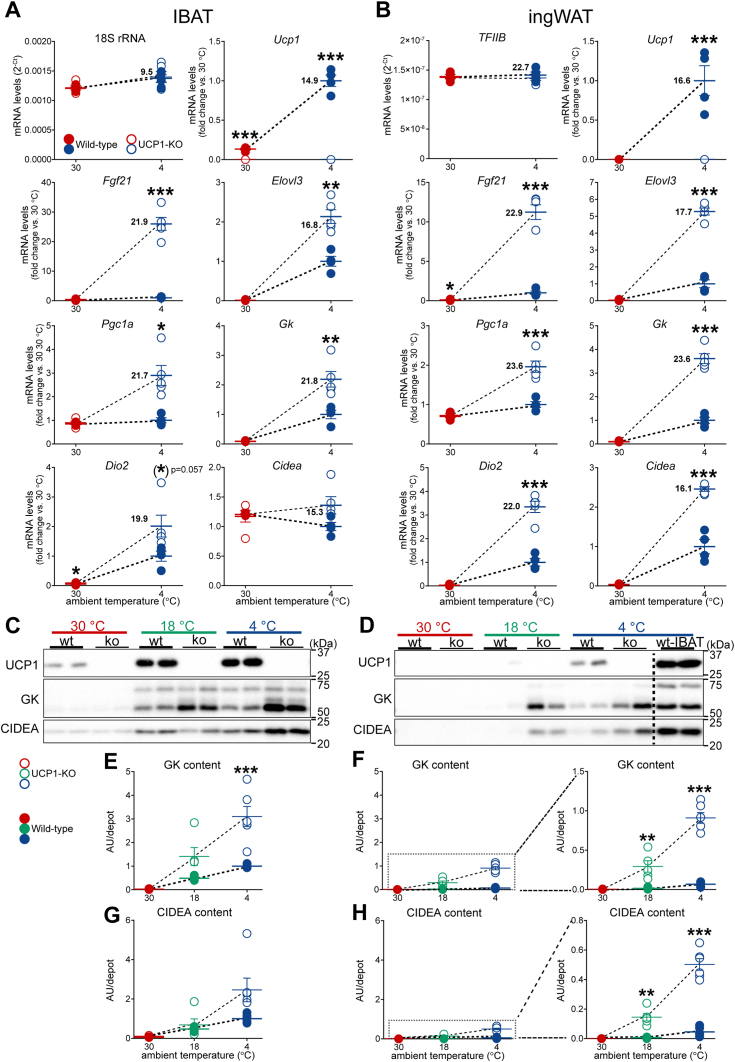
**Significant upregulation of adrenergically regulated genes in both IBAT and ingWAT of UCP1-KO mice housed below thermoneutrality.***A* and *B*, expression levels of thermogenesis-related genes in IBAT (*A*) and ingWAT (*B*) of wild-type and UCP1-KO mice acclimated to the indicated temperatures. The expression levels were normalized using reference genes: 18 S rRNA for IBAT and TFIIB for ingWAT. Each symbol represents a sample from one mouse. Values are means ± SEM. For each gene, the reported value is the mean Ct value in the condition with the highest expression. Note that Ct values were consistently higher in ingWAT than in IBAT for every examined gene, indicating lower expression levels in ingWAT. ∗Significant difference between wild-type and UCP1-KO mice for each tissue using Student’s unpaired *t* test. ∗*p* < 0.05; ∗∗*p* < 0.01; ∗∗∗*p* < 0.001. *C* and *D*, representative western blots of UCP1, glycerol kinase (GK) and CIDEA in IBAT (*A*) and ingWAT (*B*) from animals acclimated to the indicated temperatures. *E*, IBAT GK content, (*F*) ingWAT GK content, (*G*), IBAT CIDEA content, and (*H*) ingWAT CIDEA content in the indicated samples. The value in IBAT of wild-type mice acclimated to 4 °C was set to 1.0, and the levels in all other samples were expressed relative to this value. Each symbol represents a sample from one mouse. Values are means ± SEM. ∗Significant difference between wild-type and UCP1-KO mice for each tissue using two-way ANOVA followed by Tukey’s multiple comparison test. ∗∗*p* < 0.01, ∗∗∗*p* < 0.001. To facilitate comparisons of examined proteins between IBAT and ingWAT, the respective graphs were drawn with equal y-axis range. In the right panels of *D* and *F*, the graphs were redrawn with optimal y-axis range. UCP1-KO, UCP1-knockout.

Mice of both genotypes were acclimated to thermoneutrality (30 °C) or to cold (4 °C). Gene expression in IBAT and ingWAT was analyzed with real-time quantitative PCR. Reference genes were selected based on their expression, which was not affected by genotype or temperature: 18S rRNA in IBAT (Fig. 7*A*, top-left panel), and TFIIB in ingWAT (Fig. 7*B*, top-left panel) (see Experimental procedures). We initially confirmed the absence of UCP1 expression in the tissues from UCP1-KO mice (Fig. 7*A*, top-right panel) and (Fig. 7*B*, top-right panel). In line with previous data (*e.g.* (16)), acclimation to 4 °C led to highly significant cold-induced increases in UCP1 mRNA levels in both IBAT and ingWAT of wild-type mice (filled blue symbols *versus* filled red symbols). Since UCP1 mRNA could obviously not be utilized to estimate the degree of sympathetic activity in the tissues from UCP1-KO mice, we examined the extent of the induction of thermogenic marker genes known to be induced by adrenergic signaling. The genes analyzed included Fgf21, Elovl3, Pgc1a, glycerol kinase (Gk), type 2 iodothyronine deiodinase (Dio2), and Cidea. Upon acclimation to cold, the induction of practically all examined thermogenesis-related genes was significantly larger in both IBAT and ingWAT from UCP1-KO mice (Fig. 7, *A* and *B*, empty blue symbols *versus* filled blue symbols). This robust increase is strongly in accordance with an elevated sympathetic tone in these tissues. The only exception was Cidea in IBAT (Fig. 7*A*, bottom-right panel), the expression of which in brown fat is regulated posttranscriptionally and thus remained unaffected by either cold or genotype (16).

The analysis of thermogenesis-related adrenergically regulated genes at the protein level also revealed an enhanced cold-induced induction in IBAT and ingWAT from UCP1-KO mice (Fig. 7, *C*–*H*). The analysis of UCP1 again verified the absence of UCP1 expression in the tissues from UCP1-KO mice (Fig. 7*C*, top panel and Fig. 7*D*, top panel). Glycerol kinase and CIDEA protein levels (Figs. 7, *C* and *D* and S12, *A*–*D*), as well as their total contents (Fig. 7, *E*–*H*) (calculated from the data presented in Figs. 1, *C* and *D* and S12, *A*–*D*), increased in both IBAT and ingWAT of animals acclimated to 18 °C and 4 °C. However, the final protein amounts were much higher in the tissues from UCP1-KO mice compared with the tissues from wild-type mice (Fig. 7, *E*–*H*, empty symbols *versus* filled symbols). The relative increases in both glycerol kinase and CIDEA protein contents, invoked by the ablation of UCP1, were higher in ingWAT than in IBAT (Fig. 7*E* *versus* Fig. 7*F*, right panel and also Fig. 7*G* *versus*. Fig. 7*H*, right panel). It is particularly noteworthy that the amounts of both proteins in ingWAT from UCP1-KO mice acclimated to 4 °C (Fig. 7, *F* and *H*, open blue symbols) reached values similar to those in IBAT from wild-type mice also acclimated to 4 °C (Fig. 7, *E* and *G*, filled blue symbols), as was essentially the case also for tyrosine hydroxylase (see above, Fig. 6, *K* and *L*).

The observed “overinduction” of glycerol kinase and CIDEA proteins in the brown and beige adipose depots of UCP1-KO mice exposed to cold, compared to those in wild-type mice, along with the “overinduced” mRNA levels of genes known to be induced by adrenergic signaling, strongly aligns with an elevated sympathetic tone in these tissues. This suggests that the ablation of UCP1 not only led to significantly enhanced sympathetic innervation in the brown and beige adipose tissues but also resulted in a parallel increase in the activity of the sympathetic fibers that innervate these tissues. The increase in *e.g.* glycerol kinase may therefore be seen not as a compensatory effect leading to increased thermogenesis due to futile lipid cycling (*e.g.* (32, 33)) but maybe the unavoidable consequence of enhanced adrenergic stimulation.

### In UCP1-KO mice acclimated to cold, the content of mitochondrial respiratory chain proteins is strongly reduced in brown, but not in beige fat

**Figure 8:**
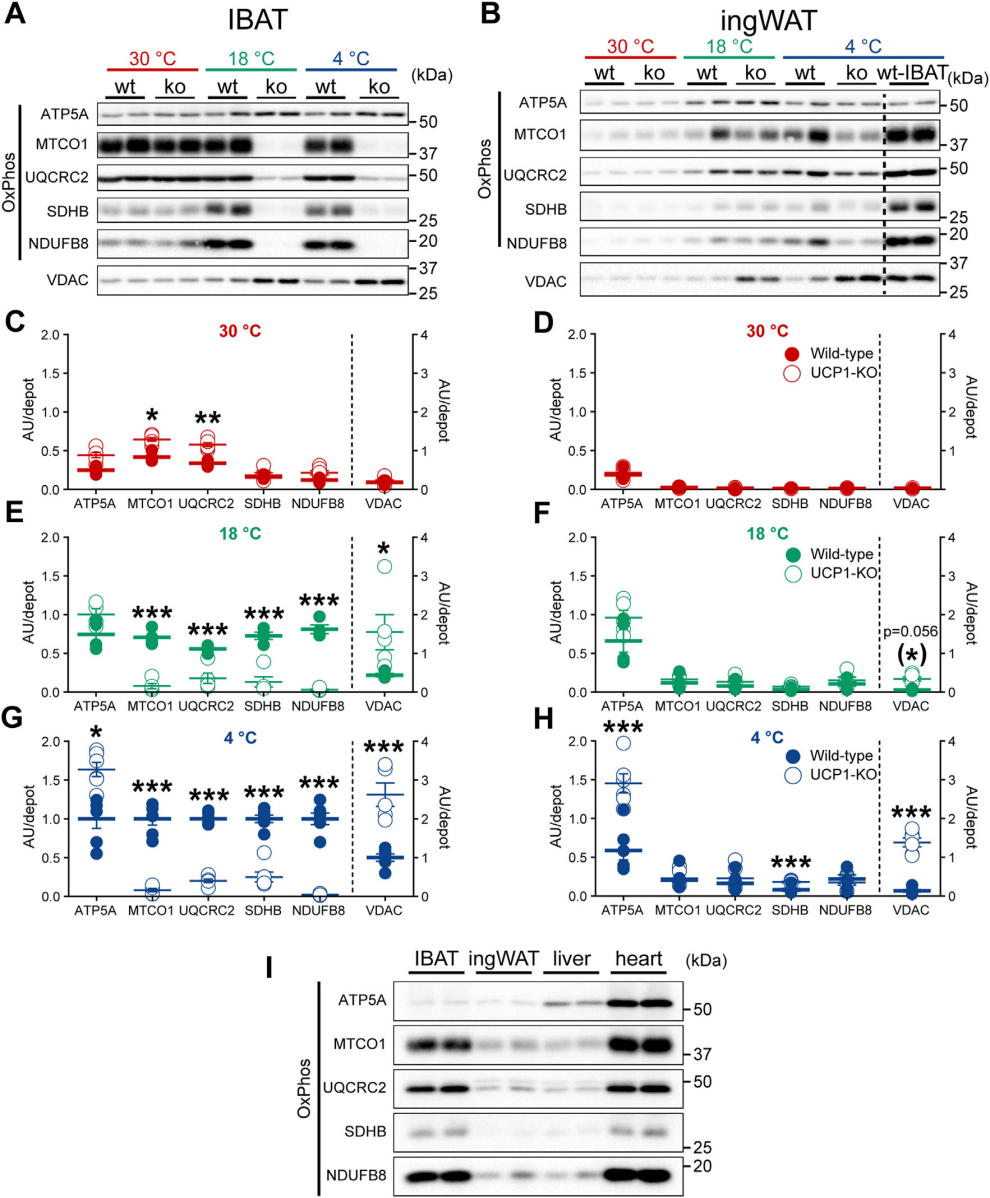
**Strongly reduced content of mitochondrial respiratory chain proteins in IBAT, but not in ingWAT, of UCP1-KO mice housed below thermoneutrality.***A* and *B*, representative western blots of mitochondrial respiratory chain proteins (ATP5A, subunit of complex V; MTCO1, subunit of complex IV; UQCRC2, subunit of complex III; SDHB, subunit of complex II; NDUFB8, subunit of complex I) and outer mitochondrial membrane protein VDAC in IBAT (*A*) and ingWAT (*B*) from animals acclimated to indicated temperatures. Note that the blots for each protein were acquired with optimal exposure. *C*–*H*, the content of the indicated mitochondrial proteins in IBAT and ingWAT from animals acclimated to 30 °C (*C* and *D*), 18 °C (*E* and *F*) and 4 °C (*G* and *H*). The mean value for each protein in IBAT of wild-type mice acclimated to 4 °C was set to 1.0, and the levels in all other samples were expressed relative to this value. Each symbol represents a sample from one mouse. Values are means ± SEM. Where not visible, the error bars are smaller than the symbols. ∗Significant difference between wild-type and UCP1-KO mice for each tissue using two-way ANOVA followed by Tukey’s multiple comparison test. ∗*p* < 0.05, ∗∗*p* < 0.01, ∗∗∗*p* < 0.001. To facilitate comparisons of examined proteins between IBAT and ingWAT and also between the three temperatures, the respective graphs were drawn with equal y-axis range. *I*, representative western blots of mitochondrial respiratory chain proteins (ATP5A, subunit of complex V; MTCO1, subunit of complex IV; UQCRC2, subunit of complex III; SDHB, subunit of complex II; NDUFB8, subunit of complex I) in IBAT, ingWAT, liver and heart tissue lysates. IBAT and ingWAT are obtained from wild-type mice acclimated to 4 °C; liver and heart are obtained from wild-type mice acclimated to 21 °C. Note that whereas the subunits of respiratory complexes I – IV were expressed at similar levels in the tissues with high oxidative capacity - IBAT and heart, ATP5A was an exception by being expressed at much lower levels in IBAT (as well as in ingWAT). UCP1-KO, UCP1-knockout.

Mitochondriogenesis represents an essential part of cold-induced recruitment of thermogenic capacity in brown and beige adipose tissues (*e.g.* (16, 34)). This is of special importance as previous studies reported a notable global mitochondrial derangement and remarkably low levels of respiratory chain subunits in brown fat from UCP1-KO mice (3, 4, 5); ingWAT was not studied but our earlier study indicated normal respiratory function in ingWAT mitochondria deficient in UCP1 (34) (in agreement with (11)). To detail whether or not an adequate mitochondriogenesis was a component of cold-induced recruitment in IBAT and ingWAT lacking UCP1, we measured the levels of mitochondrial proteins - representative subunits of the respiratory complexes I–V and VDAC (Fig. 8, *A*–*H*).

The content of specific mitochondrial proteins in the whole adipose depots was calculated by multiplying the protein levels of these mitochondrial proteins expressed per mg tissue protein (Fig. S13) with the total protein content in the tissue (Fig. 1, *C* and *D*). For each of the analyzed proteins, the values in IBAT of wild-type mice acclimated to 4 °C were set to 1.0 (Fig. 8*G*, blue filled symbols), and the levels in all other samples were expressed relative to this value. The data sets for each of the acclimation temperatures and each of the tissues are presented in separate graphs (Fig. 8, *C*–*H*), ordered from top to bottom with the data for 30 °C presented at the top (red symbols), for 18 °C in the middle (green symbols) and for 4 °C at the bottom (blue symbols); the data sets for IBAT are presented on the left and those for ingWAT on the right.

As expected, in IBAT from wild-type mice, the content of all examined mitochondrial proteins increased with exposure to increased cold stress; the values were lowest in the IBAT from the mice acclimated to 30 °C (Fig. 8*C*, red filled symbols) and were significantly increased with exposure to 18 °C (Fig. 8*E*, green filled symbols) and were even further increased with the exposure to 4 °C (Fig. 8*G*, blue filled symbols). A similar increase in the content of specific mitochondrial proteins was observed in beige adipose tissue (compare Fig. 8*D* (red-filled symbols), 8F (green-filled symbols) and 8H (blue-filled symbols)). This thus verified the gradually increased thermogenic recruitment in both tissues. Importantly, the content of each analyzed mitochondrial protein in beige adipose tissue was much lower compared with brown adipose tissue at all acclimation temperatures (Fig. 8, *D*, *F* and *H* *versus* Fig. 8, *C*, *E* and *G*); ATP5A was an exception by being similar in the two tissues.

Analysis of UCP1-KO brown fat demonstrated that the contents of proteins belonging to respiratory chain complexes I - IV in the cold-acclimated mice were indeed dramatically diminished compared to those in wild-type brown fat (Fig. 8, *E* and *G*, empty *versus* filled symbols), in agreement with (3). However, Kazak *et al.* (3) also reported a similar but less pronounced reduction of proteins belonging to respiratory chain complexes I - IV in brown fat of UCP1-KO mice acclimated to thermoneutrality. In contrast to this, we found that under thermoneutral conditions all examined mitochondrial proteins in UCP1-deficient brown fat were largely unaffected (or even increased) (Fig. 8*C*) (in agreement with (5, 35, 36)). The content of VDAC was increased in IBAT from UCP1-KO mice (Fig. 8, *C*, *E* and *G*) and thus could not be used for the estimation of mitochondrial number.

In contrast, in beige adipose depots from wild-type and UCP1-KO mice, the levels of proteins belonging to respiratory chain complexes I - IV (Figs. 8*B* and S13, *B*, *D* and *F*) displayed far less drastic differences than in brown fat (Figs. 8*A* and S13, *A*, *C* and *E*). The only proteins expressed at lower levels in UCP1-KO ingWAT than in ingWAT from wild-type mice were the representative subunits of respiratory complexes I and IV, only in animals acclimated to 4 °C (Fig. S13*F*). However, importantly, the content of all representative subunits of respiratory complexes in the entire ingWAT depot was similar in the two genotypes (Fig. 8, *D*, *F* and *H*) (in agreement with our earlier study (34)).

The proteins belonging to complex V (ATP5A) and the outer mitochondrial membrane (VDAC) displayed a different expression profile; similarly to the case in brown fat, the contents of ATP5A and VDAC were significantly increased also in ingWAT from UCP1-KO mice acclimated to subthermoneutral temperatures (Fig. 8, *F* and *H*) (also in agreement with (34)).

Importantly, a distinctive characteristic of both brown and beige-fat mitochondria *versus*, *e.g.*, heart mitochondria, is a remarkably low content of the ATP synthase (ATP5A) ((34, 37, 38, 39, 40) and Fig. 8*I*). This molecular feature of brown and beige-fat mitochondria is functionally reflected in their limited ability to produce ATP through oxidative phosphorylation (34). The implication of this, even having considered the increase in ATP synthase content in UCP1-deficient mitochondria, is that ATP-consuming mechanisms are unlikely to be responsible for major alternative (UCP1-independent) thermogenic processes in brown and beige adipocytes.

In summary, we confirm here that in the cold, the absence of UCP1 in brown adipose tissue resulted in remarkably diminished levels of respiratory chain subunits in brown-fat mitochondria but demonstrate importantly that this is not the case in beige fat. The cold-induced increase in protein content in UCP1-KO brown fat, not different from that in wild-type mice (Fig. 1*C*), was not due to an increase in mitochondrial content and thus the tissue was not in a recruited state; the significant accumulation of macrophages (Fig. 2) may partially account for the increase in the total tissue protein observed in the brown fat of UCP1-KO mice acclimated to cold (Fig. 1*C*). Considering the observed decrease in respiratory chain subunits and the increase in macrophage accumulation, it could be suggested that IBAT from the UCP1-KO mice acclimated to cold had undergone atrophy. In contrast to brown fat, beige adipose tissue had undergone an enhanced recruitment: the mitochondria would seem to be unaffected and the tissue displayed an almost 3-fold increased protein content and lower adiposity (Fig. 1*D*).

## Discussion

In the cold, the ablation of UCP1 results in hyper-recruitment of beige fat but the brown fat contrastingly becomes atrophied. Here, we attempt to reveal the mechanisms underlying this phenomenon. We revealed that the macrophages that massively accumulated not only in IBAT but also in ingWAT of the UCP1-KO mice acclimated to cold were *not* equipped with tyrosine hydroxylase, and also lacked NET and MAO-A and thus could not influence the tissues through the synthesis or degradation of norepinephrine. Thus, adipose tissue norepinephrine is exclusively of sympathetic origin. Notably, sympathetic innervation in both tissues was significantly increased, and proteins known to be induced by adrenergic stimulation displayed a parallel increase in their total tissue amounts. In agreement with this, the beige adipose depot from the UCP1-KO mice acclimated to cold displayed an enhanced recruitment state which thus may be seen as a canonical consequence of the enhanced innervation of the tissue. However, the *magnitude* of sympathetic innervation and also the expression levels of glycerol kinase, CIDEA, and MAO-A in the two tissues were markedly different, being much higher (2–10-fold) in brown fat. Consequently, the atrophy of the UCP1-deficient brown fat may be seen as a consequence of supraphysiological adrenergic stimulation in this tissue. The atrophy could potentially be mediated by an increase in MAO-A and resulting oxidative stress.

### The macrophages that massively accumulate in IBAT and ingWAT of the UCP1-KO mice acclimated to cold do not influence the tissues by modulating norepinephrine availability

Macrophages represent the major immune cell types residing in adipose tissues (41, 42). Under obesogenic conditions, macrophages massively infiltrate *visceral* white adipose tissue (WAT) (43, 44); they aggregate around dead or dying adipocytes and form multinucleate crown-like structures (45, 46). Compared to visceral WAT, *subcutaneous* WAT displays a much lower propensity to accumulate macrophages (*e.g.* (47)). Also brown fat can accumulate remarkably high amounts of macrophages but the main factor leading to this is very prolonged exposure to thermoneutrality (19). However, under subthermoneutral conditions, both brown and beige (subcutaneous) fat demonstrate negligible amounts of macrophages. In this study, we detailed a unique (patho)physiological situation: a massive macrophage accumulation into both brown and beige fat that occurs in UCP1-KO mice under subthermoneutral conditions.

Certain macrophages, some of which are referred to as SAMs (sympathetic neuron–associated macrophages), found in brown and beige fat, have been suggested to play an important role in modulating norepinephrine availability and thus also a role in modulating thermogenic activity of these tissues (21, 24). Adipose tissues from the UCP1-KO mice acclimated to cold thus provided us with an unprecedented opportunity to further examine the competence of adipose tissue macrophages to modulate norepinephrine availability. However, we found that these macrophages did *not* express tyrosine hydroxylase, NET, and MAO-A, and consequently, they were *not* competent to either synthesize or degrade norepinephrine. Thus, the vast majority of macrophages accumulated within UCP1-deficient brown and beige fat under cold conditions cannot affect the tissues by modulating norepinephrine availability. These macrophages can thus not explain the diametrically opposite response to the cold of UCP1-deficient brown *versus* beige fat.

The conclusion that the macrophages massively accumulated in UCP1-deficient adipose tissues upon prolonged exposure to cold do *not* modulate norepinephrine availability in these tissues evidently raises the question of the physiological function of these macrophages. We suggest that they execute their conventional (but probably not their only) function: phagocytosis and degradation of dead cells, dying cells, and cellular debris. This scenario implies a high degree of cell death or a high degree of cell damage in adipose tissues from the UCP1-abated mice exposed to subthermoneutral conditions (supported by unpublished results of Kramarova L, Shabalina I and Boulet N). The nature of the signal(s) that triggers macrophage accumulation is not known, but some hypothetical signals are discussed below.

The possible role of adipose tissue-resident macrophages in the control of tissue innervation (48), the possible presence of cholinergic adipose macrophages (ChAMs) (49), and the ‘identity’ of macrophages—M1 *versus* M2 subtype at a single-cell level – have not been investigated here.

### A possible physiological explanation for the high abundance of sympathetic nerves

Thermogenic recruitment—the increase in the capacity of thermogenesis-competent adipose depots to perform thermogenesis upon acclimation to cold—is mediated by norepinephrine (50). In this study, we demonstrated that the adipose tissue norepinephrine is exclusively of sympathetic origin. Exposure to subthermoneutral temperatures results in signals from the hypothalamus to increase sympathetic activity to brown (50) and also to beige adipose tissue (51, 52), in order to initiate recruitment. With time, this recruitment will result in an enhanced capacity for heat production, through enhanced cell proliferation and differentiation. A certain degree of sympathetic tone, proportional to the cold intensity, will maintain the recruited state once the necessary capacity has been reached. We suggest that the same process occurs in UCP1-KO mice—a given cold stress will activate the sympathetic outflow to brown and beige adipose tissues. As these tissues are unable to produce the heat required, the sympathetic activity will become further intensified, in a vain attempt by the central mechanisms of the animal to generate a heat-producing tissue. Thus, at any degree of cold stress, the tissues are more adrenergically stimulated than they are in the corresponding tissues of the wild-type mice.

In addition to the central mechanisms, adipose afferent sensory neurons have been also proposed to play a role in modulating sympathetic outflow to adipose tissues (53, 54). Similarly, sensory neurons within ingWAT may act as an inhibitory brake on the local sympathetic function (55). Therefore, also through these mechanisms, the lack of heat production in the UCP1-deficient adipose tissues would likely result in an augmentation of the local sympathetic activity.

### Assessment of sympathetic activity

The remarkably increased expression levels of the norepinephrine-synthesizing enzyme tyrosine hydroxylase in both brown and beige fat of UCP1-KO mice, along with the unexpectedly acquired ability of brown and beige adipocytes to express the NE-degrading enzyme MAO-A, suggest an increased NE-turnover and thus also an increased adrenergic stimulation in IBAT and ingWAT from the UCP1-KO mice acclimated to cold. To estimate the magnitude of sympathetic activity in these tissues, we used an indirect approach: we measured mRNA and protein levels of adipose-selective molecular marker genes known to be induced by adrenergic signaling. The expression levels of these adrenergically induced genes correlate positively with the expression levels of tyrosine hydroxylase and thus strongly suggest a parallel increase in sympathetic activity.

The norepinephrine turnover rate and thus the sympathetic tone has been assessed in brown fat from the wild-type and UCP1-KO mice acclimated to 20 to 22 °C (56) (beige fat has not been examined). Sympathetic activity, even under these mild cold conditions, was about 8-fold higher in the IBAT of the UCP1-KO mice than that of the wild-type mice. Thus, our conclusion regarding the sympathetic activity in adipose tissues from UCP1-KO *versus* wild-type mice, based on an indirect approach, is in full agreement with the data obtained by measuring norepinephrine turnover rate.

### Hyper-recruitment of beige fat

Higher adrenergic innervation in beige adipose tissue from UCP1-KO mice would be expected to result in hyper-recruitment of the tissue. In agreement with this expectation, the beige adipose depot from the UCP1-KO mice acclimated to the cold displayed enhanced recruitment (Fig. 1, *B*, *D* and *F*). This enhanced recruitment may therefore be understood not as a compensatory effect leading to UCP1-independent thermogenic responses, but rather as an unavoidable consequence of the enhanced adrenergic stimulation to the tissue because of the absence of thermogenesis.

In recent years, a number of alternative adaptive thermogenic mechanisms independent of UCP1 have been suggested. These have included futile cycles in a broad sense, such as a lipolysis/re-esterification cycle (*e.g.* (5, 32, 33)), creatine cycling (57), and calcium cycling (58). All these suggested mechanisms may be classified as being ATP-dependent, since they require that ATP is formed and then used in an ‘unproductive’ way, leading to ADP generation and consequently to stimulation of substrate oxidation/oxygen consumption in the mitochondria (*i.e.*, thermogenesis). Thus, thermogenic mechanisms that are independent of UCP1 can only be accomplished in tissues that are endowed with considerable phosphorylation capacity, and their presence would be best discerned in tissues devoid of UCP1. The UCP1-KO mice thus represent an excellent tool to examine both UCP1-dependent and UCP1-independent thermogenic mechanisms. In the beige adipose depot deficient in UCP1, the oxidative capacity, compared with that of wild-type mice, is not diminished, although still much lower than in brown fat (Fig. 8 and (34)). Even having considered the increase in ATP synthase in the beige fat from UCP1-KO mice (Fig. 8), beige mitochondria generally have a remarkably low ATP synthase capacity (Fig. 8*I* and (34)). This would then indicate that ATP-consuming mechanisms are not likely to be responsible for major thermogenic processes in beige adipocytes, not even in UCP1-KO mice.

### Why does sympathetic hyper-innervation not result in hyper-recruitment of brown fat?

In UCP1-KO mice acclimated to cold, sympathetic innervation was remarkably increased in both brown and beige adipose tissue. However, in contrast to the beige adipose tissue that, as expected, had undergone a hyper-recruitment, brown fat had become atrophied. Even though the increase in the total tissue protein in UCP1-KO mice acclimated to cold was not different from that in wild-type mice and thus appeared to indicate proper tissue recruitment (Fig. 1*C*), the content of mitochondrial respiratory chain proteins was strongly reduced (in agreement with (3, 4, 5)), the degree of adiposity was apparently higher and the tissue was massively infiltrated with macrophages. This may be interpreted to indicate that the recruitment of brown fat is not primarily regulated by sympathetic activity. However, as the *magnitude* of sympathetic innervation and also the expression levels of genes known to be induced by adrenergic signaling (Fig. 7) and MAO-A were much higher in brown than in beige fat, a more plausible alternative would be that the brown fat from UCP1-KO mice acclimated to cold is under such strong adrenergic stimulation that it could be considered to be beyond physiological.

Under strong cold stimulation, adrenergically induced lipolysis results in the release of fatty acids. In wild-type mice, these fatty acids are mostly shuttled towards the mitochondria where they initiate thermogenesis by activating UCP1 and are also utilized as a substrate for thermogenesis. Since the brown adipocytes lacking uncoupling activity have a limited capacity to combust fatty acids, it may be hypothesized that in brown fat deficient in UCP1, the free fatty acids reach such a high level that this results in cytotoxic effects. Whether this scenario, alone or in combination with oxidative stress caused by MAO-A (see below), could underlie the remarkable decrease in respiratory chain proteins in UCP1-KO tissues, remains to be investigated. Immune cells that massively accumulate into UCP1-deficient brown fat upon exposure to cold (Fig. 2 and (3, 7)) may be an indication of the tissue’s defense mechanism in response to *e.g.* lipolysis-(cold)-induced cytotoxicity or to damage caused by ROS produced due to MAO-A activity.

### An impressive induction and unexpected adipocyte localization of MAO-A

The biological effects of the norepinephrine released from the sympathetic nerves are terminated rapidly by norepinephrine being taken up into the sympathetic nerve endings and/or to effector cells, or by conversion of norepinephrine into inactive metabolites (28). Such a role in taking up and degrading norepinephrine has been ascribed to specialized adipose tissue macrophages (those equipped with NET and MAO-A, referred to as SAMs, as discussed above). Such macrophages could not be identified here among the macrophages that were very abundantly accumulated in UCP1-deficient adipose tissues upon exposure to cold. In contrast, a large number of *adipocytes*, both brown and beige, displayed high expression of MAO-A (Figs. 4 and 5). The regulatory mechanisms underlying this unexpectedly acquired ability of mouse adipocytes to express MAO-A remain to be identified. Importantly, these remarkably high levels of the NE-degrading enzyme MAO-A correlate positively with the expression levels of the NE-producing enzyme tyrosine hydroxylase and thus may reflect the need for lowering norepinephrine levels in adipose tissues of the UCP1-KO mice acclimated to cold.

MAO-A is a flavoenzyme localized to the outer mitochondrial membrane that generates H_2_O_2_ as a by-product of norepinephrine catabolism (59, 60). Thus, MAO-A is a potent generator of reactive oxygen species (ROS). MAO-A is often implicated in neurological and cardiovascular diseases, and it is mainly investigated in these organs. In the heart, catecholamine degradation by MAO-A (and also by MAO-B) has been recognized as a significant source of mitochondrially produced ROS (*e.g.* (61)). During post-myocardial infarction remodeling, the sustained sympathetic drive and chronically elevated circulating catecholamines fuel MAO-A activity and ROS generation; the subsequent cardiolipin peroxidation results in 4-hydroxynonenal (HNE) production inside the mitochondria. MAO-A-derived HNE forms adducts with VDAC and the mitochondrial calcium uniporter (MCU). This, in turn, causes mitochondrial Ca^2+^ overload, mitochondrial respiratory dysfunction, and loss of membrane potential (62). Brown fat from the UCP1-KO mice acclimated to cold displays some analogy with the post-infarction heart. As shown in the current study, it is characterized by massively enhanced sympathetic innervation, abundantly expressed MAO-A and a strongly reduced content of mitochondrial respiratory chain proteins. Furthermore, UCP1-deficient BAT mitochondria exhibit reduced mitochondrial calcium buffering capacity and are highly sensitive to mitochondrial permeability transition induced by ROS and calcium overload (3). This analogy makes us hypothesize that the strongly reduced content of mitochondrial respiratory chain proteins could be the consequence of the oxidative stress caused by the intensive MAO-A-mediated norepinephrine degradation and ROS production. Although our earlier study did not reveal a general increase in HNE/protein adducts in brown-fat mitochondria isolated from UCP1-KO mice (35), it is important to note that our study did not specifically examine HNE adducts on certain proteins, such as VDAC and MCU.

Similarly to the case in brown fat, beige fat from the UCP1-KO mice acclimated to cold is characterized by an enhanced sympathetic innervation and remarkably high MAO-A expression. Despite this, the mitochondrial respiratory chain proteins in this tissue were largely unaffected. We propose that the reason for this is that the levels of both sympathetic innervation and MAO-A in UCP1-deficient beige fat were still much lower than in brown fat (about 2-5-fold).

Another hypothetical function of MAO-A, based on analogies with dopaminergic neurons (63) and Leydig cells in prematurely aging mice (64), could be to transfer electrons to the electron transport chain (most likely at the level of cytochrome c), and in this way support mitochondrial energization and bypass respiratory chain dysfunction. Due to the strongly reduced content of mitochondrial respiratory chain proteins, this pathway would be more relevant in brown than in beige fat from the UCP1-KO mice acclimated to the cold.

## Conclusion

We demonstrate here that no qualitative differences in innervation or macrophage characteristics can explain the divergent reactions of classical brown *versus* beige adipose tissues to UCP1-ablation. Accordingly, we suggest that quantitative differences in sympathetic innervation suffice to explain the distinction, in that sympathetic hyper-innervation in brown fat leads to cytotoxic reactions, possibly mediated by augmented ROS production from MAO-A.

## Experimental procedures

### Animals

All experiments were approved by the Animal Ethics Committee of the North Stockholm region. UCP1-KO mice were the progeny of those described in (1), backcrossed to the C57Bl/6J background. The mice were bred and maintained in-house as homozygous lines (UCP1-knockout and wild-type). To avoid genetic drift, UCP1-knockout and wild-type lines were regularly intercrossed. The mice were fed *ad libitum* (Labfor R70; Lantmännen), had free access to water, and were kept on a 12:12 h light:dark cycle. At the end of the experiments, animals were sacrificed using CO_2_ anesthesia. IBAT and ingWAT were quantitatively dissected. The left and right lobes were placed in separate tubes. They were either directly snap-frozen in liquid nitrogen and stored at −80 °C for subsequent Western blot or qPCR analysis, or fixed in formaldehyde solution for histological examination.

*Cohort 1*, used for Western blot and histological analysis, consisted of mice acclimated to 30 °C, 18 °C and 4 °C. Mice that were acclimated to 4 °C remained in their original cages at 21 °C until 6 weeks of age. Mice were then single-caged and transferred to 18 °C for 2 weeks and then to 4 °C for the following 8 weeks. Mice that were acclimated to 18 °C and to 30 °C, remained in their original cages at 21 °C until 10 weeks of age. Then, the mice were single-caged and directly transferred to 30 °C or to 18 °C, where they remained for 6 weeks. At the end of acclimation (termination of the experiments), all mice were 16 weeks old. The number of animals used was as follows: wild-type at 30 °C, n = 4; wild-type at 18 °C, n = 4; wild-type at 4 °C, n = 6; UCP1-KO at 30 °C, n = 6; UCP1-KO at 18 °C, n = 5; UCP1-KO at 4 °C, n = 6.

*Cohort 2*, used for mRNA analysis, consisted of mice acclimated to 30 °C and 4 °C. Mice remained in their original cages at 24 °C until 7 to 8 weeks of age. Mice were then single-caged and acclimated to thermoneutrality (30 °C) or, in parallel, successively acclimated to 4 °C by first being placed at 18 °C for 1 week and then at 4 °C for the following 7 weeks. At the end of acclimation (termination of the experiment), all mice were 15 to 16 weeks old. The number of animals used was as follows: wild-type at 30 °C, n = 4; wild-type at 4 °C, n = 4; UCP1-KO at 30 °C, n = 6; UCP1-KO at 4 °C, n = 6.

### Protein analysis

#### Sample processing and protein quantification

Frozen tissues were homogenized in a modified RIPA buffer (50 mM Tris·HCl, pH 7.4, 1% Triton X-100, 150 mM NaCl, 1 mM EDTA) with freshly added 1 mM Na_3_VO_4_, 10 mM NaF and protease inhibitor cocktail (Complete-Mini, Roche) at a specific ratio, typically 1:10 (w/vol). The homogenates, after freezing (in liquid nitrogen) and subsequent defrosting to ensure complete lysis of adipose cells, were centrifuged at 14,000*g* for 15 min. The top fat layer was discarded, and the lysate (infranatant) was carefully aspirated using a 1 ml syringe and 27 G needle.

The protein concentration in the lysate was determined using the Lowry method. The total protein content in the depot was calculated by multiplying the protein concentration (in μg protein/μl lysate) by the dilution factor and the wet tissue weight.

#### Western blot analysis

An equal volume of reducing sample buffer (125 mM Tris·HCl, pH 6.8, 4% (wt/vol) SDS, 20% (vol/vol) glycerol, 100 mM dithiothreitol, and 0.1% (wt/vol) bromphenol blue) was added to each sample. Equal amounts of protein were separated by SDS-PAGE in ordinary 12% polyacrylamide gel (acrylamide/bis-acrylamide = 37.5/1) or high-resolution 12% polyacrylamide gel (acrylamide/bis-acrylamide = 175/1) (OxPhos proteins and glycerol kinase). Proteins were transferred to polyvinylidene difluoride membranes (BioRad) in 48 mM Tris·HCl, 39 mM glycine, 0.037 (wt/vol) SDS and 15% (vol/vol) methanol, using a semi-dry electrophoretic transfer cell (Bio-Rad Trans-Blot SD; Bio-Rad) at 1.2 mA/cm^2^ for 90 min. After washing, the membrane was blocked in 5% milk in Tris-buffered Saline-Tween for 1 h at room temperature and probed with the indicated antibodies overnight at 4 °C. The immunoblot was visualized with appropriate horseradish peroxidase-conjugated secondary antibodies and enhanced chemiluminescence (Clarity Western ECL Substrate, Bio-Rad) in a charge-coupled device camera (ChemiDoc XRS+, Bio-Rad). Analysis of the blots was performed using Image Lab 6.1 software (Bio-Rad). Samples loaded on different membranes were compared by normalization of band intensity with a standard sample of brown fat loaded on all membranes.

Antibodies used in the western blots were: MAC-2 (Santa Cruz Laboratories, sc-23938), diluted 1:5000; MAO-A (Abcam, ab126751), diluted 1:2000; tyrosine hydroxylase (Abcam, ab137869), diluted 1:2000; UCP1 (rabbit polyclonal, raised against C-terminal decapeptide), diluted 1:15,000; glycerol kinase (Abcam, ab126599), diluted 1:2000; CIDEA (Santa Cruz Biotechnology, sc-366814), diluted 1:500; OxPhos Rodent WB Antibody cocktail (Invitrogen, 458099), diluted 1:10,000; VDAC (Cell Signaling Technology, 4661S), diluted 1:2000; β-actin (Invitrogen, MA1-140), diluted 1:5000; β-tubulin (Invitrogen, MA5-16308), diluted 1:2000; FABP4 (Cell Signaling Technology, 2120S), diluted 1:2000. Western blot data were not normalized to housekeeping proteins because in the very plastic IBAT and ingWAT we were not able to identify a protein that displays constant expression across different experimental conditions and different tissues. Therefore, normalization was maintained by equal sample loading. However, as a number of the examined proteins were either not expressed or were expressed at very low levels in some of the samples, successful loading as such was verified by probing membranes with β-actin, β-tubulin or FABP4 antibodies, or, after the results were acquired, membranes were stained with Amido black.

#### Immunohistochemistry

Immunohistochemistry was performed principally as in (19). In Figures 2, 3 and 6, antibodies raised in different species were employed which thus enabled simultaneous multiplex staining. In Figures 4 and 5, antibodies from the same host species (rabbit) were employed and therefore sequential multiplex staining was performed (principally as described in (65)). Slides were kept in the dark after secondary antibody incubation. Sections were analyzed in a confocal Zeiss LSM 780 microscope (Carl Zeiss Micro Imaging). For optimal visualization of each of the samples, the images were acquired with settings allowing in each case the maximum signal detection below the saturation limits of the detectors. Consequently, due to differing exposure times for each sample, the images captured cannot be directly compared quantitatively.

A detailed description of immunohistological analyses is provided in the Supporting information.

### mRNA analysis

#### RNA isolation and cDNA synthesis

Frozen tissues were homogenized in TRI Reagent (T9424; Sigma-Aldrich), and the chloroform-isopropanol method was used to isolate RNA according to the Sigma-Aldrich TRI Reagent protocol. The RNA concentrations in the samples were measured with a Thermo Scientific NanoDrop One Spectrophotometer. The High-Capacity cDNA Reverse Transcription Kit (Cat. No. 4368814; Applied Biosystems) was used to reverse transcribe 500 ng of total RNA into cDNA in a total volume of 20 μl. After the reaction was completed, cDNA was diluted 10 times in water.

#### Real-time qPCR

All primers were validated before use to ensure good amplification efficiency (90–110%) and specificity (controlled for by melting curve analysis and inclusion of control samples in which the reverse transcriptase had been left out of the reaction). Gene-specific primers (see Table S2) and SYBR Green JumpStart Taq Ready Mix (S4438; Sigma-Aldrich) were premixed in a total volume of 11 μl. The final primer concentration used was 0.3 μM. Two microliters of the diluted cDNA were added to the premixed primer solution to a total volume of 13 μl. All samples were run in triplicate. The Bio-Rad CFX Connect Real-Time system was used to perform the real-time quantitative polymerase chain reaction. The samples were preheated 2 min at 50 °C and 10 min at 95 °C, after which 40 cycles of 15 s at 95 °C and 1 min at 60 °C were run. The real-time qPCR reaction was followed by melting curve analysis.

The ΔCt method was used to calculate relative changes in mRNA abundance. Ct values for 18S rRNA (for IBAT samples, Figs. 7*A* and S5) or for general transcription factor IIb (TFIIB) (for ingWAT samples, Fig. 7*B*) were subtracted from the Ct values of each analyzed gene (ΔCt method) to adjust for variability in cDNA synthesis. These ΔCt values were antilog-transformed (2^−ΔCt^) to determine changes in mRNA abundance. Reference gene expression was analyzed as 2^−Ct^ and was generally similar among samples (see Fig. 7*A*, top left panel and Fig. 7*B*, top left panel).

## Data availability

All data are contained within the article and supporting information.

## Supporting information

This article contains supporting information (65, 66).

## Conflict of interest

The authors declare that they have no conflicts of interest with the contents of this article.
