# Supplementary material for: Differential responses to UCP1 ablation in classical brown versus beige fat, despite a parallel increase in sympathetic innervation
Source: J Biol Chem. 2024 Feb 16;300(3):105760. doi: 10.1016/j.jbc.2024.105760 (PMC10944106; doi:10.1016/j.jbc.2024.105760)
Supplement: Supporting Information [file mmc1.pdf]

SUPPORTING INFORMATION

Differential responses to UCP1 ablation in classical brown versus beige fat, despite a parallel increase in sympathetic innervation

Qimuge Naren[1,2], Erik Lindsund[2], Muhammad Hamza Bokhari[2], Weijun Pang[1,*], Natasa Petrovic[2,*]

Detailed immunohistochemistry protocol

The right lobe/pad of the adipose tissue was immersion-fixed in 4 % alcoholic formaldehyde (4 % formaldehyde in ethanol) for 24 hours. It was then dehydrated and embedded in paraffin using a standard procedure [1]. Tissues were sectioned using a standard microtome (Leica RM2255, Leica Microsystems). The sections, each 5 µm thick, were mounted on SuperFrost® Plus adhesion slides (VWR International bvba, Leuven, Belgium). Subsequently, the sections were deparaffinized and rehydrated. To unmask antigenicity, the deparaffinized and rehydrated slides were boiled in citrate buffer (10 mM sodium citrate, pH 6) in a water bath for 30 minutes. After boiling, they were cooled on the benchtop for another 30 minutes. In order to block auto-fluorescence, the sections were then incubated in 0.3 % Sudan Black B (Sigma-Aldrich, 199664) in 70 % ethanol for 30 minutes at room temperature. Following this step, the slides were rinsed with PBS and placed in a humid chamber for a 2-hour incubation with a blocking solution (3 % BSA in PBS) at room temperature. Negative controls were included to detect auto-fluorescence and any nonspecific binding. For the primary antibody incubation, the primary antibodies were diluted in 1 % BSA in PBS. A volume of 50-100 µl was pipetted onto each tissue section, and they were then incubated for 24 hours at 4 °C in a humid chamber.

Simultaneous multiplex staining

In Figures 2, 3 and 6, antibodies raised in different species were employed which thus enabled simultaneous multiplex staining. Antibodies used were:

Figure 2: perilipin (rabbit monoclonal) (Cell Signaling Technology, 4661S), diluted 1:250 and MAC-2 (rat monoclonal) (Santa Cruz Laboratories, sc-23938), diluted 1:100.

Figure 3: tyrosine hydroxylase (rabbit monoclonal) (Abcam, ab137869) diluted 1:100, MAC-2 (Santa Cruz Laboratories, sc-23938), diluted 1:100 and perilipin (goat ployclonal) (Abcam, ab61682), diluted 1:100.

Figure 6: tyrosine hydroxylase (Abcam, ab137869), diluted 1:100 and perilipin (goat polyclonal) (Abcam, ab61682), diluted 1:100.

After the primary antibody incubation, the slides were washed with PBS for 1 h and then incubated with secondary antibodies diluted in 1 % BSA in PBS; a volume of 50-100 µl was pipetted onto each tissue section, followed by a 2-hour incubation at room temperature in a humid chamber. Secondary antibodies were:

Figure 2: chicken anti-rat Alexa Fluor 488-labelled secondary antibody (Molecular Probes, A21470), diluted 1:100; goat anti-rabbit Alexa Fluor 594-labelled secondary antibody (Molecular probes, A11037), diluted 1:250.

Figure 3: chicken anti-rabbit Alexa Fluor 488-labelled secondary antibody (Molecular Probes, A21441), diluted 1:100, chicken anti-rat Alexa Fluor 594-labelled secondary antibody (Molecular Probes, A21471), diluted 1:100 and donkey anti-goat Alexa Fluor 647-labelled secondary antibody (Molecular probes, A21447), diluted 1:100.

Figure 6: chicken anti-rabbit Alexa Fluor 488-labelled secondary antibody (Molecular Probes, A21441), diluted 1:100; donkey anti-goat Alexa Fluor 594-labelled secondary antibody (Molecular probes, A11058), diluted 1:100.

After secondary antibody incubation, sections were washed with PBS for 1 h. To stain nuclei, the sections were incubated in 1 µg/ml Hoechst 33258 (Sigma-Aldrich, 861405) for 10 min, washed with PBS for 30 min and mounted with ProGold® antifade reagent (Molecular Probes, P36934). Slides were kept in the dark after secondary antibody incubation. Sections were analyzed in a confocal Zeiss LSM 780 microscope (Carl Zeiss Micro Imaging).

Sequential multiplex staining

In Figures 4 and 5, antibodies from the same host species (rabbit) were employed and therefore sequential multiplex staining was performed (principally as described in [2]). Tyramide signal amplification (TSA) kits (Thermo Fisher Scientific, B40943 and/or B40944) were employed in the process. Prior to the blocking, the endogenous peroxidase activity was quenched by adding 3 % hydrogen peroxide solution (Component C2 from the kit) and incubated for 1 h at room temperature. The slides were washed with PBS for 1 h, followed by blocking with BSA. Following this, the slides were incubated with primary antibodies. Antibodies used were:

Figure 4A,B: MAO-A (rabbit monoclonal) (Abcam, ab126751), diluted 1:150; perilipin (rabbit monoclonal) (Cell Signaling Technology, 4661S), diluted 1:250 and MAC-2 (rat monoclonal) (Santa Cruz Laboratories, sc-23938), diluted 1:100.

The slides were initially incubated with the MAO-A antibody, overnight. Following the primary antibody incubation, the slides were washed with PBS for 1 h. Then, the slides were incubated with a poly-HRP-conjugated secondary antibody (Component B) for 1 h at room temperature. After this, the slides were washed with PBS for 1 h. Next, the slides were incubated with a tyramide working solution (labelled with Alexa Fluor 594) for a duration of 10 minutes, following the instructions provided in the kit. Following the incubation, the slides were again washed with PBS. To confirm the successful staining, the slides were examined under an epifluorescence microscope. The tyramide fluorescence label is covalently bound to the proteins in the sample and, it is also thermally stable. This allowed for the subsequent removal of both the primary and secondary antibodies from the sample by boiling the slides in a citrate buffer. The slides were boiled in the citrate buffer (10 mM sodium citrate, pH 6) in a water bath for 30 minutes. After boiling, the slides were allowed to cool on the benchtop for 30 minutes. After cooling, the samples were incubated with BSA solution for 30 min. Following this, the samples were

simultaneously incubated with MAC-2 and perilipin antibodies. Subsequent to this point, the same protocol as described above was employed. Secondary antibodies were: chicken anti-rat Alexa Fluor 488-labelled secondary antibody (Molecular Probes, A21470), diluted 1:100 and goat anti-rabbit Alexa Fluor 647-labelled secondary antibody (Molecular Probes, A21245), diluted 1:250.

Figure 4C,D: NET (rabbit monoclonal) (Abcam, ab ab254361), diluted 1:100, tyrosine hydroxylase (Abcam, ab137869), diluted 1:100 and MAC-2 (Santa Cruz Laboratories, sc-23938), diluted 1:100. The slides were initially incubated with the NET antibody, overnight. Once the staining for NET was completed (with Alexa Fluor 594-labelled tyramide), the slides were boiled, cooled and blocked (as described above). Next, the slides were incubated with the tyrosine hydroxylase and MAC-2 antibodies. Following this, the same protocol as in Figure 3A,B was employed. Secondary antibodies were: chicken anti-rat Alexa Fluor 488-labelled secondary antibody (Molecular Probes, A21470), diluted 1:100 and goat anti-rabbit Alexa Fluor 647-labelled secondary antibody (Molecular Probes, A21245) diluted 1:100.

Figure 5: MAO-A (rabbit monoclonal) (Abcam, ab126751), diluted 1:150; tyrosine hydroxylase (rabbit monoclonal) (Abcam, ab137869), diluted 1:150 and perilipin (rabbit monoclonal) (Cell Signaling Technology, 4661S), diluted 1:250. The slides were initially incubated with the tyrosine hydroxylase antibody, overnight. Once the staining for tyrosine hydroxylase was completed (with Alexa Fluor 488-labelled tyramide), the slides were boiled, cooled and blocked (as described above). Next, the slides were incubated with the MAO-A antibody and stained using Alexa Fluor 594-labeled tyramide. After this step, the process was repeated (the slides were again boiled, cooled and blocked). Finally, the slides were incubated with the perilipin antibody. Perilipin was visualized using the secondary antibody: goat anti-rabbit Alexa Fluor 647-labelled secondary antibody (Molecular Probes, A21245) diluted 1:250.

References

[1] Cinti, S., Zingaretti, M.C., Cancello, R., Ceresi, E., Ferrara, P., 2001. Morphologic techniques for the study of brown adipose tissue and white adipose tissue. Methods Mol Biol 155:21-51.

[2] Toth, Z.E., Mezey, E., 2007. Simultaneous visualization of multiple antigens with tyramide signal amplification using antibodies from the same species. J Histochem Cytochem 55(6):545-554.

Table S1. Two-way ANOVA P values for temperature, genotype and interaction.

	IBAT			ingWAT		
	Temperature	Genotype	Interaction	Temperature	Genotype	Interaction
Tissue weight	0.0130*	0.0002*	0.2220	0.0220*	<0.0001*	0.0635
Protein content	<0.0001*	0.1269	0.1971	<0.0001*	<0.0001*	<0.0001*
Protein density	<0.0001*	0.0001*	0.0001*	<0.0001*	0.0003*	0.0004*
MAC-2 content	<0.0001*	<0.0001*	<0.0001*	0.0046*	<0.0001*	0.0007*
MAC-2 per mg prot.	0.0001*	<0.0001*	<0.0001*	0.1075	0.0004*	0.1504
MAO-A content	0.0006*	<0.0001*	0.0022*	<0.0001*	<0.0001*	<0.0001*
MAO-A per mg prot.	0.0079*	<0.0001*	0.0048*	<0.0001*	<0.0001*	0.0002*
TH content	<0.0001*	<0.0001*	<0.0001*	<0.0001*	0.0001*	<0.0001*
TH density	0.0005*	0.0009*	0.0151*	<0.0001*	<0.0001*	<0.0001*
TH per mg prot.	0.0056*	0.0002*	0.0129*	<0.0001*	<0.0001*	<0.0001*
GK content	<0.0001*	0.0001*	0.0020*	<0.0001*	<0.0001*	<0.0001*
GK per mg prot.	<0.0001*	<0.0001*	<0.0001*	<0.0001*	<0.0001*	<0.0001*
CIDEA content	<0.0001*	0.0381*	0.0597	<0.0001*	<0.0001*	0.0001*
CIDEA per mg prot.	<0.0001*	0.0184*	0.0077*	<0.0001*	<0.0001*	<0.0001*
ATP5A content	<0.0001*	0.0008*	0.2965	<0.0001*	<0.0001*	0.0011*
ATP5A per mg prot.	<0.0001*	0.0001*	0.0190*	<0.0001*	0.9090	0.7985
MTCO1 content	0.0085*	<0.0001*	<0.0001*	<0.0001*	0.4625	0.8609
MTCO1 per mg prot.	<0.0001*	<0.0001*	<0.0001*	0.0007*	0.1479	0.0734
UQCRC2 content	<0.0001*	<0.0001*	<0.0001*	0.0004*	0.2417	0.7022
UQCRC2 per mg prot.	<0.0001*	<0.0001*	<0.0001*	0.0010*	0.4835	0.1513
SDHB content	<0.0001*	<0.0001*	<0.0001*	<0.0001*	<0.0001*	0.0003*
SDHB per mg prot.	0.0003*	<0.0001*	<0.0001*	<0.0001*	0.4974	0.0925
NDUFB8 content	<0.0001*	<0.0001*	0.0001*	0.0001*	0.9583	0.4957
NDUFB8 per mg prot.	<0.0001*	<0.0001*	<0.0001*	0.0013*	0.1215	0.0355*
VDAC content	<0.0001*	<0.0001*	0.0059*	<0.0001*	<0.0001*	<0.0001*
VDAC per mg prot.	<0.0001*	<0.0001*	<0.0001*	<0.0001*	<0.0001*	<0.0001*

Values were calculated as described in Materials and methods. IBAT, interscapular brown adipose tissue; ingWAT, inguinal white adipose tissue. * P values <0.05. The number of animals used was: wild-type at 30 °C, n=4; wild-type at 18 °C, n=4; wild-type at 4 °C, n=6; UCP1-KO at 30 °C, n=6; UCP1-KO at 18 °C, n=5; UCP1-KO at 4 °C, n=6.

Table S2. Primer sequences

Gene	Forward (5' - 3')	Reverse (5' - 3')
Cx3cr1	AAGTTCCCTTCCCATCTGCT	CAAAATTCTCTAGATCCAGTTCA
Adgre1	GGAGGACTTCTCCAAGCCTATT	AGGCCTCTCAGACTTCTGCTT
Lgals3	GCCTACCCCAGTGCTCCT	GGTCATAGGGCACCGTCA
Cd68	CGGTGGAATACAATGTGTCCT	GATGAATTCTGCGCCATGA
Fcgr1	TGTGGCTTCTAACAACTCTGCT	ACAGCCTTGGTGGCATTAAC
Tnfa	TCTTCTCATTCCTGCTTGTGG	GGTCTGGGCCATAGAACTGA
Ccl2	CATCCACGTGTTGGCTCA	GATCATCTTGCTGGTGAATGAGT
Mrc1	CAAACTGGGGGAAAGGCTAT	TTGCCACCAATCACAACTACA
Mrc2	TACAGCTCCACGCTATGGATT	CACTCTCCCAGTTGAGGTACT
Clec10a	TGAGAAAGGCTTTAAGAACTGG	GACCACCTGTAGTGATGTGGG
Spp1	CCCGGTGAAAGTGACTGATT	ATCTGGGTGGAGGCTGTAA
Pgc1a	GAAAGGGCCAAACAGAGAGA	GTAAATCACACGGCGCTCTT
Ucp1	GGCCTCTACGACTCAGTCCA	TAAGCCGGCTGAGATCTTGT
Dio2	CTGCGCTGTGTCTGGAAC	GGAATTGGGAGCATCTTCAC
Elovl3	GCCTCTCATCCTCTGGTCCT	TGCCATAAACTTCCACATCCT
Cidea	GCCTGCAGGAACTTATCAGC	AGAACTCCTCTGTGTCCACCA
Gk	CCGCGAAGAAAGCAGTTCTG	CAAAAAACGTGTCGAGCTGGT
Fgf21	AGATGGAGCTCTCTATGGATCG	GGGCTTCAGACTGGTACACAT
18S rRNA	AGTCCCTGCCCTTTGTACACA	CGATCCGAGGGCCTCACTA
TFIIB	TGGAGATTTGTCCACCATGA	GAATTGCCAAACTCATCAAAACT

Figure S1

IBAT - UCP1-KO - 4 °C

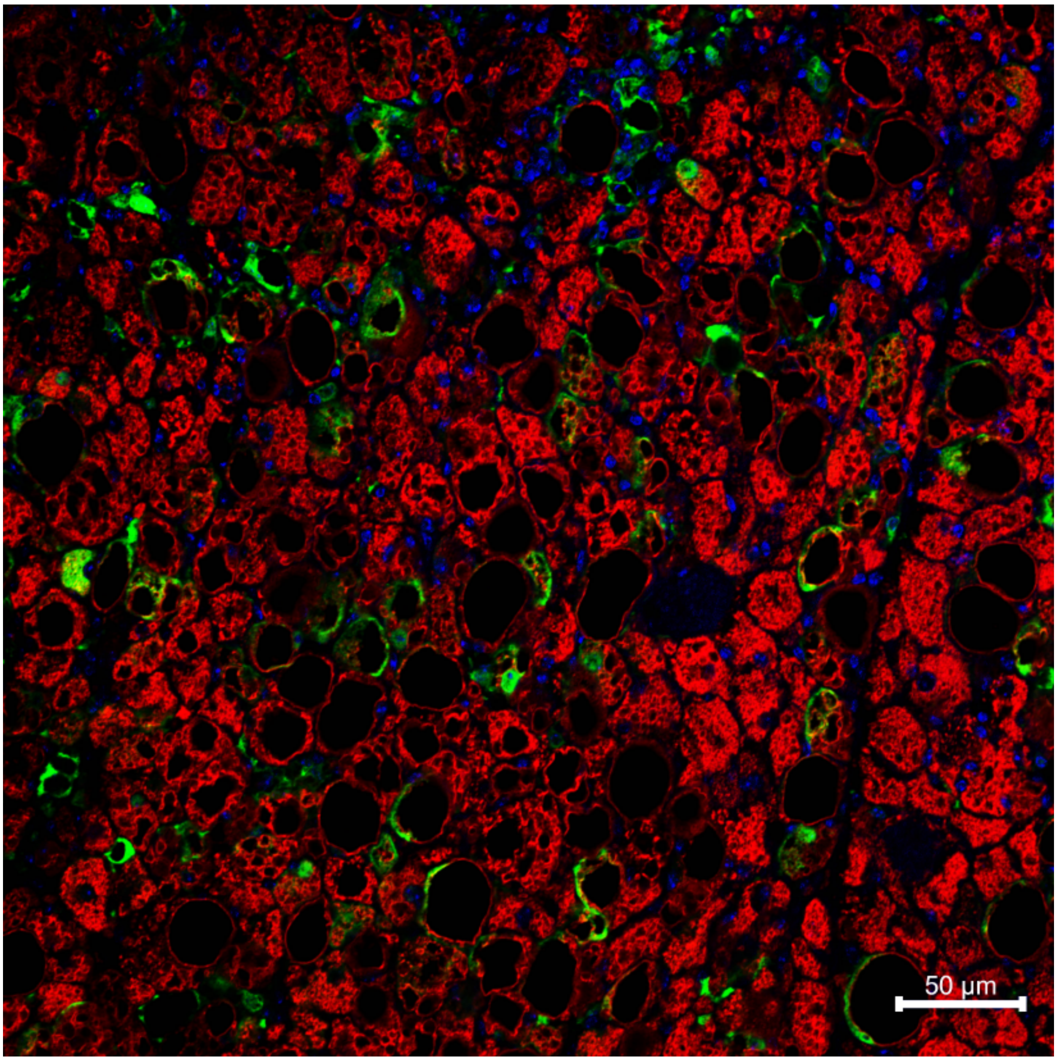

Green: MAC-2 Red: perilipin Blue: nuclei

Figure S1. A notable accumulation of macrophages in IBAT of UCP1-KO mice acclimated to 4 °C. Enlarged version of Figure 2J. The tissue was stained for MAC-2 (green), perilipin (red) and nuclei (blue). Scale bar 50 µm.

Figure S2

ingWAT - UCP1-KO - 4 °C

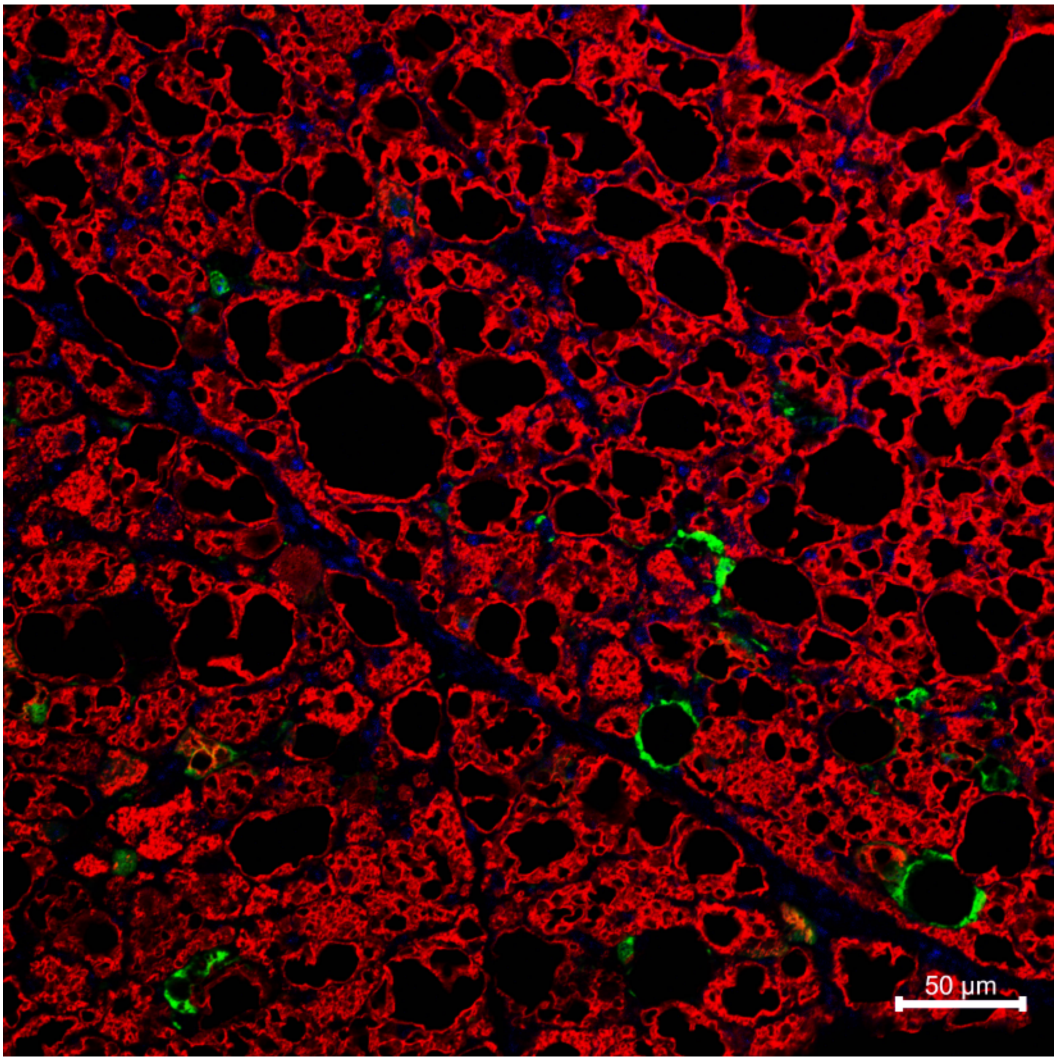

Green: MAC-2 Red: perilipin Blue: nuclei

Figure S2. A notable accumulation of macrophages in ingWAT of UCP1-KO mice acclimated to 4 °C. Enlarged version of Figure 2L. The tissue was stained for MAC-2 (green), perilipin (red) and nuclei (blue). Scale bar 50 µm.

Figure S3

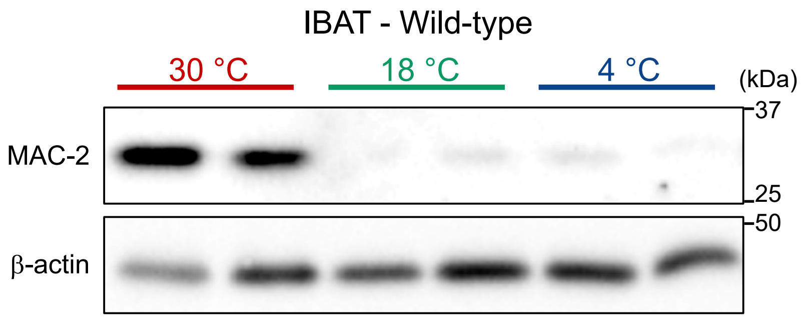

Figure S3. An increased presence of macrophages in IBAT from wild-type mice under thermoneutral conditions. Western blot of MAC-2 in IBAT from wild-type animals acclimated to the indicated temperatures (the same animals as in Figure 2M). When brown fat samples from thermoneutral mice are analyzed in parallel with the samples from UCP1-KO mice acclimated to subthermoneutral temperatures, MAC-2 is undetectable in the samples from the thermoneutral mice (Figure 2M). This observation can be attributed to the relative difference in MAC-2 levels between thermoneutral brown fat and brown fat from UCP1-KO mice acclimated to subthermoneutral temperatures, which surpasses the linear dynamic range of MAC-2 protein detection. Consequently, within this specific experimental setup, the levels of MAC-2 in thermoneutral brown fat are below the detection limit for MAC-2, consistent with immunostaining results depicted in Figure 2F and J). However, when analyzing samples only from wild-type mice, the MAC-2 protein is readily detected in the brown fat of thermoneutral mice.

Figure S4

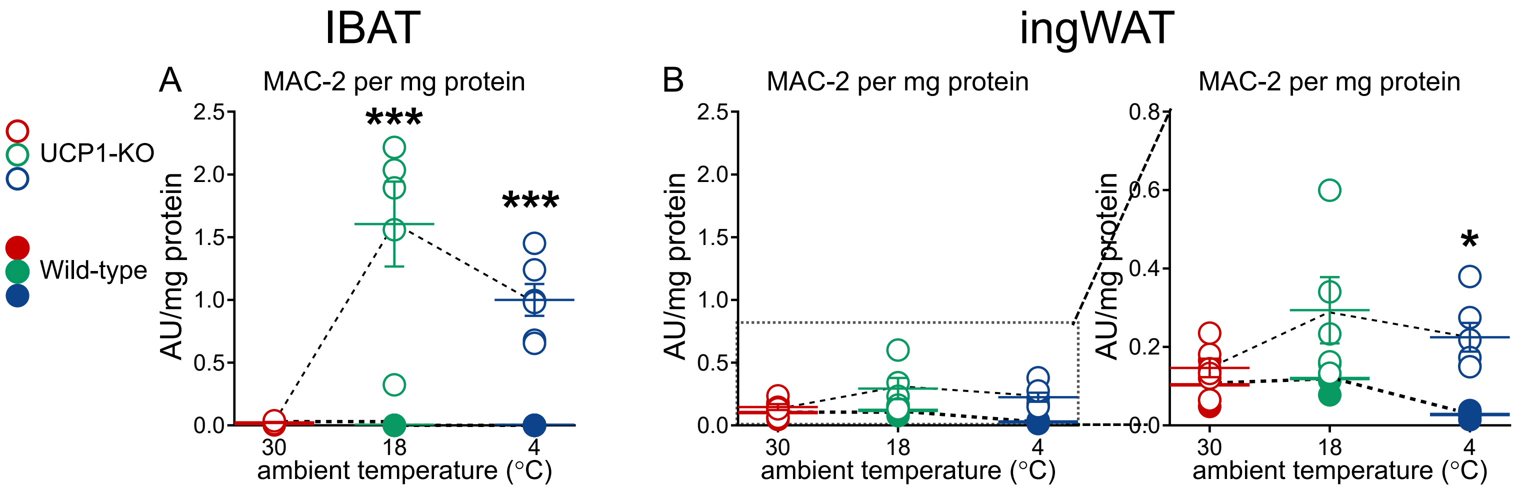

Figure S4. (A-B) Protein levels (quantification of immunoblots) of MAC-2 in IBAT (A) and ingWAT (B) of the same animals as in Figures 1-8. The mean value in IBAT of UCP1-KO mice acclimated to 4 °C was set to 1.0, and the levels in all other samples were expressed relative to this value. Each symbol represents a sample from one mouse. Values are means ± SEM. Where not visible, the error bars are smaller than the symbols. Full ANOVA statistics are given in Table S1. *Significant difference between wild-type and UCP1-KO mice for each tissue using two-way ANOVA followed by Tukey's multiple comparison test. *P < 0.05, ***P < 0.001. To facilitate comparisons of MAC-2 levels between IBAT and ingWAT, the respective graphs were drawn with equal y-axis range. In the right panel of B, the graph was redrawn with optimal y-axis range.

Figure S5

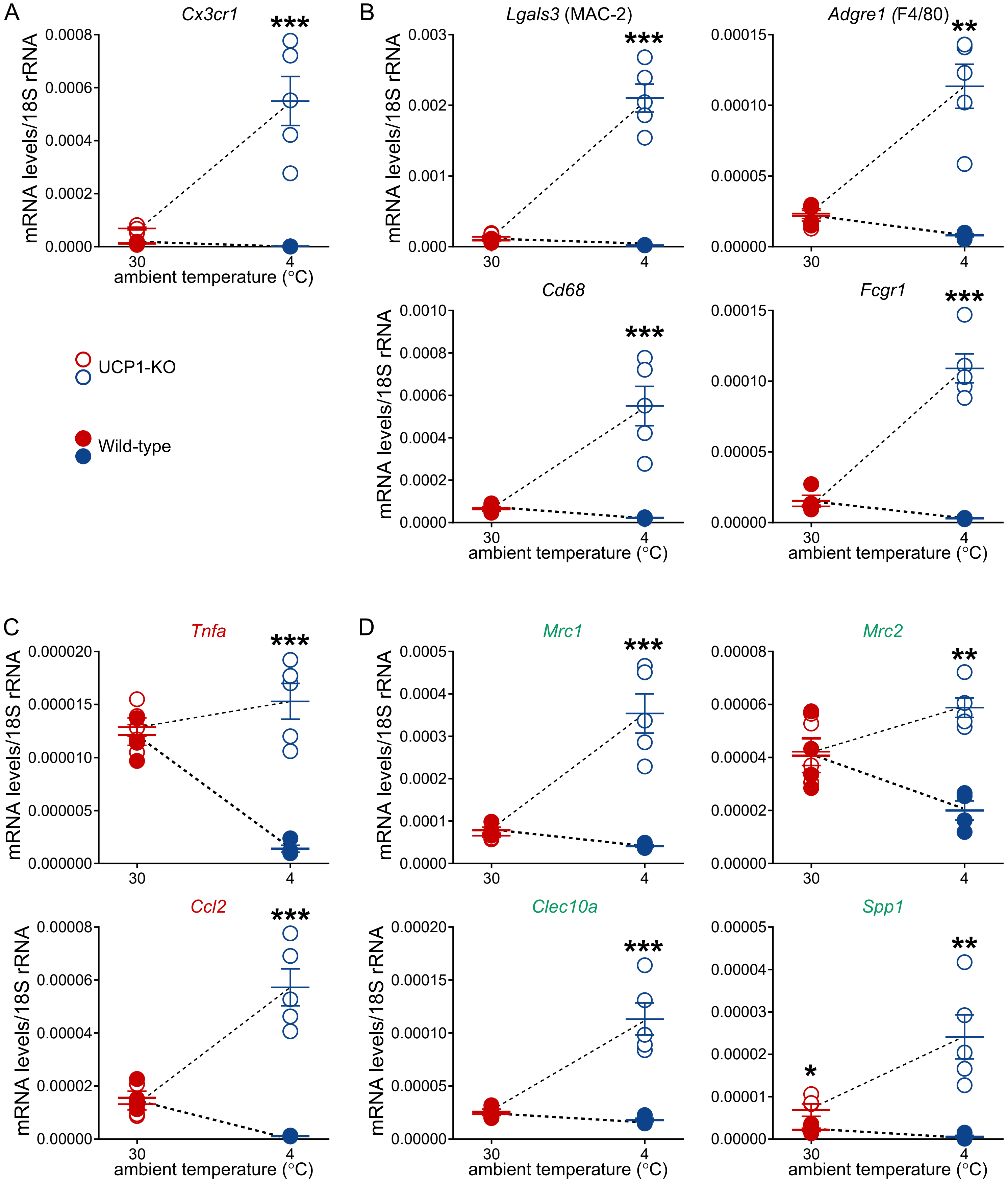

Figure S5. A significant increase in the expression of macrophage marker genes in IBAT of UCP1-KO mice acclimated to cold. (A-D) Gene expression levels of tissue-resident macrophage marker gene (A), general macrophage marker genes (B), M1 macrophage marker genes (C) and M2 macrophage marker genes (D) in IBAT of wild-type and UCP1-KO mice acclimated to thermoneutrality (n = 4) or to cold (4 °C) (n = 5). Each symbol represents a sample from one mouse. Values are means ± SEM. Where not visible, the error bars are smaller than the symbols. *Significant difference between wild-type and UCP1-KO mice using Student's unpaired t-test. *P < 0.05; **P < 0.01; ***P < 0.001.

Figure S6

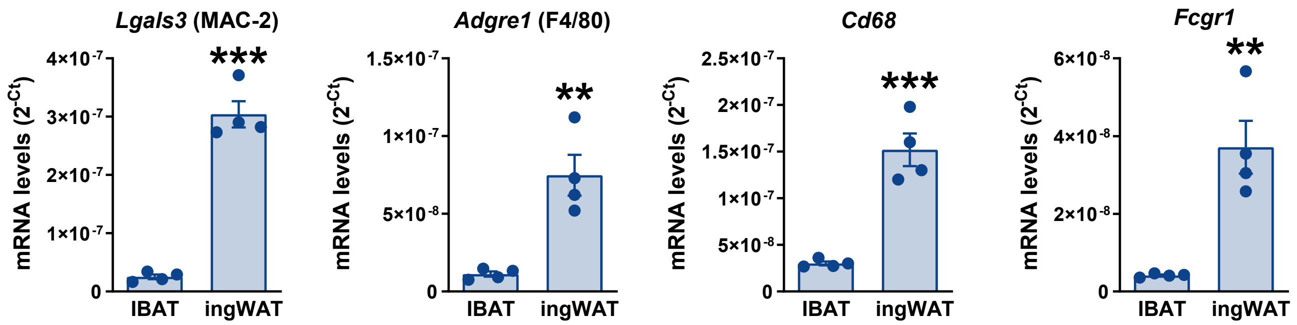

Figure S6. Gene expression levels of general macrophage marker genes in IBAT (n=4) and ingWAT (n=4) of wild-type mice acclimated to 4 °C. Values are antilog-transformed Ct values (2^{-Ct}) presented as means ± SEM. *Significant difference between IBAT and ingWAT using Student's unpaired t-test. **P < 0.01, ***P < 0.001.

Figure S7

IBAT - UCP1-KO - 4 °C

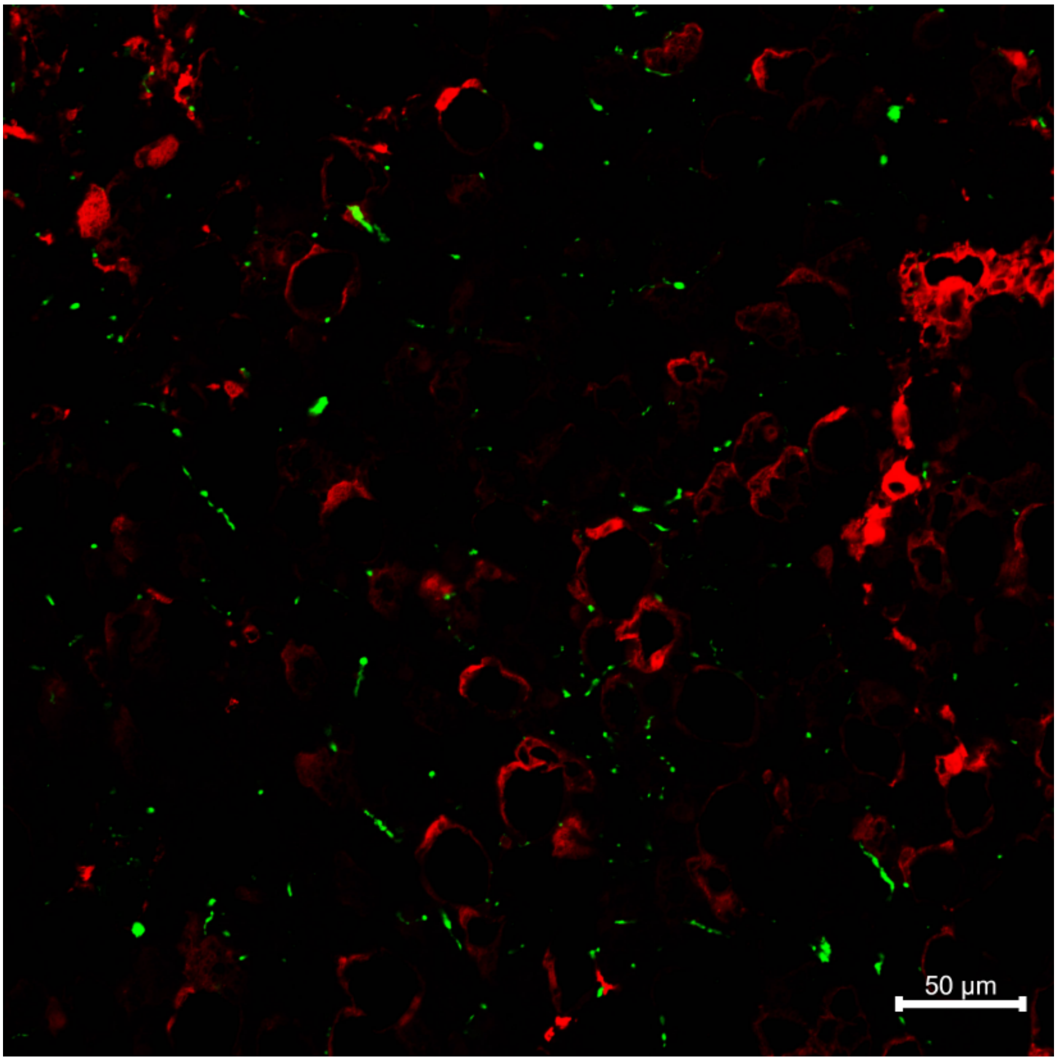

Green: TH Red: MAC-2

Figure S7. Macrophages within IBAT of UCP1-ablated mice acclimated to 4 °C do not express tyrosine hydroxylase (TH). Enlarged version of Figure 3C. The tissue was stained for TH (green) and MAC-2 (red). Scale bar 50 μm.

Figure S8

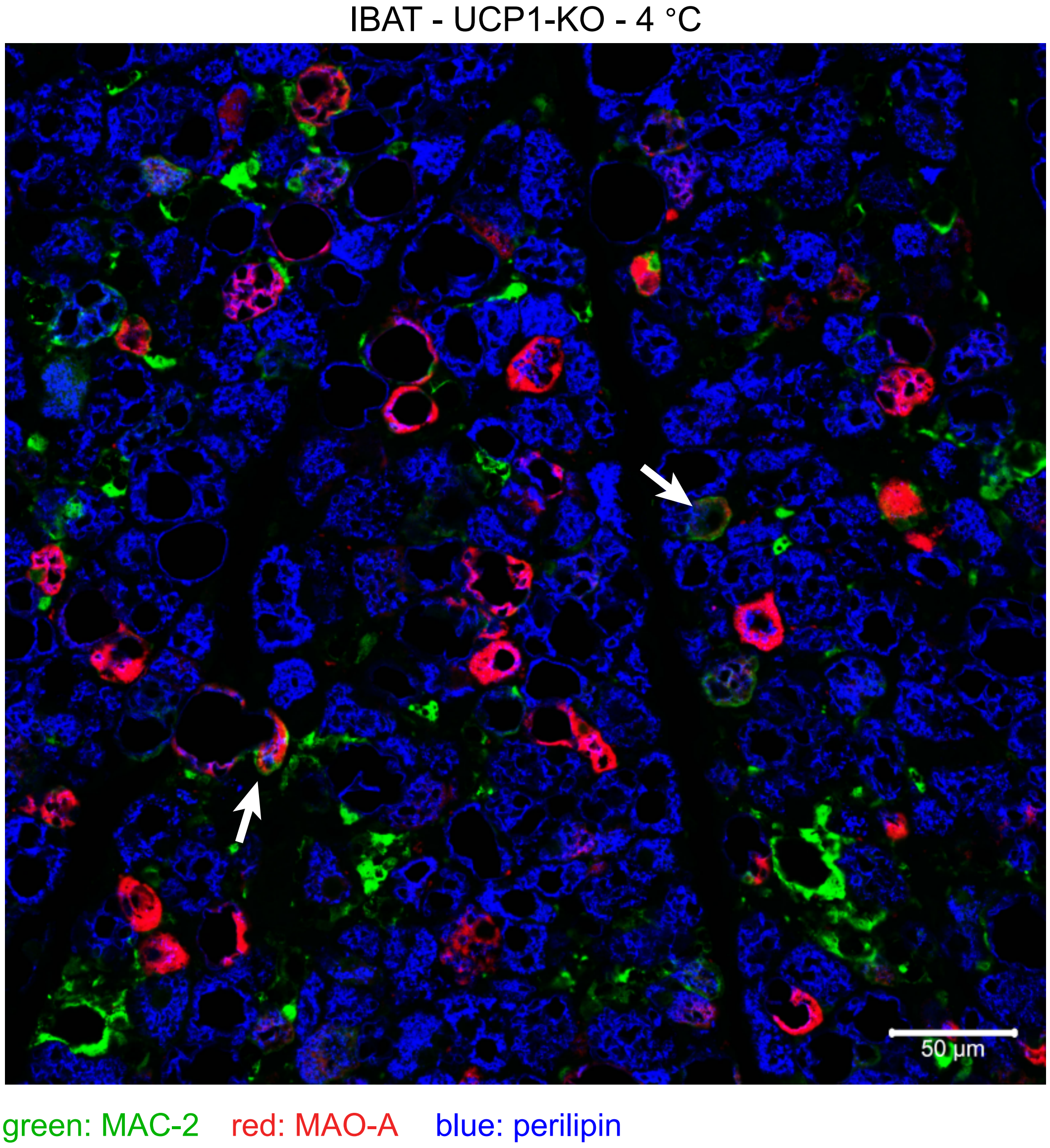

Figure S8. Macrophages within IBAT of UCP1-ablated mice acclimated to 4 °C do not express MAO-A. Enlarged version of Figure 4A. The tissue was stained for MAC-2 (green), MAO-A (red) and perilipin (blue). Cells displaying an ambiguous staining pattern are depicted with white arrows. Scale bar 50 µm.

Figure S9

IBAT - UCP1-KO - 4 °C

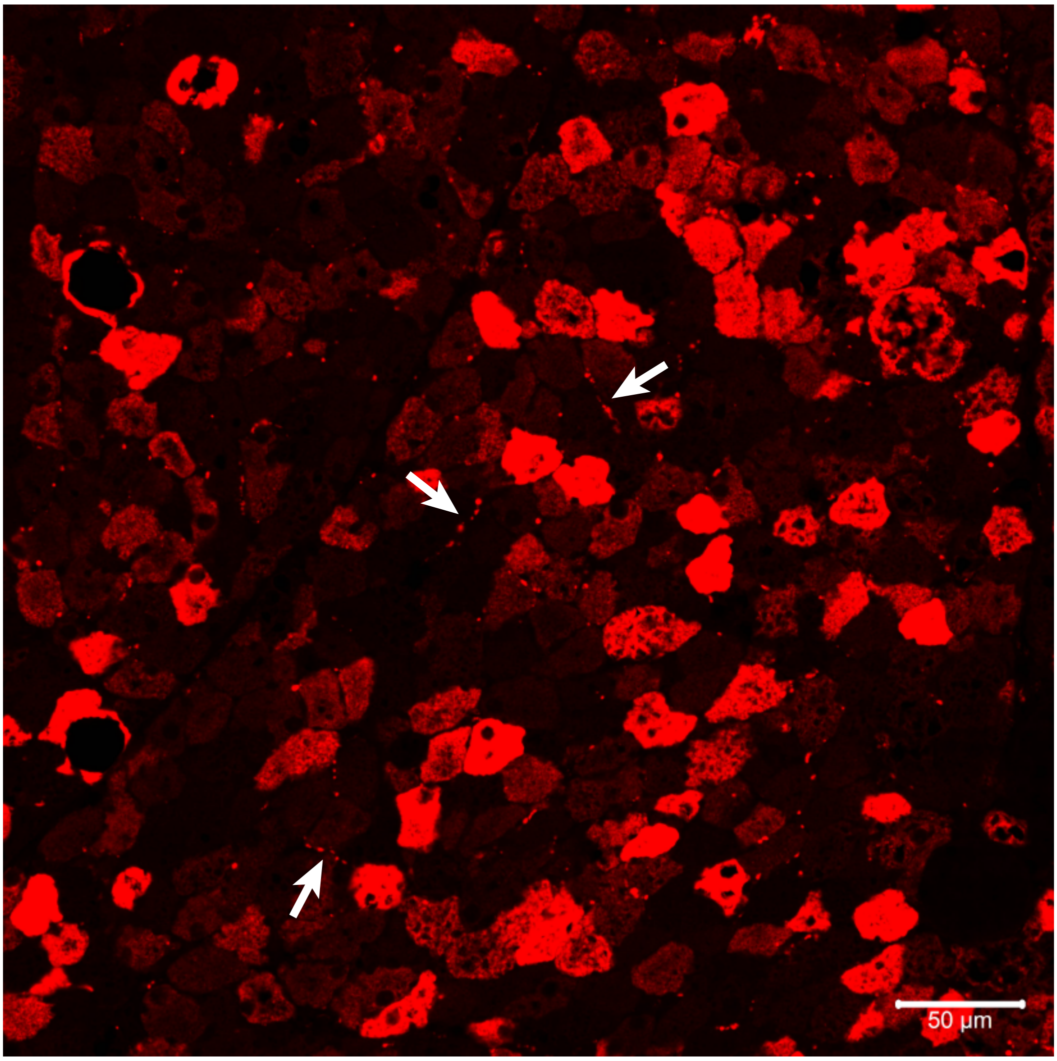

red: MAO-A

Figure S9. Both adipocyte and sympathetic localization of MAO-A within IBAT of UCP1-KO mice acclimated to 4 °C. Overexposed and enlarged version of Figure 5B. The tissue was stained for MAO-A (red). The examples of sympathetically localized MAO-A are depicted with white arrows. Scale bar 50 µm.

Figure S10

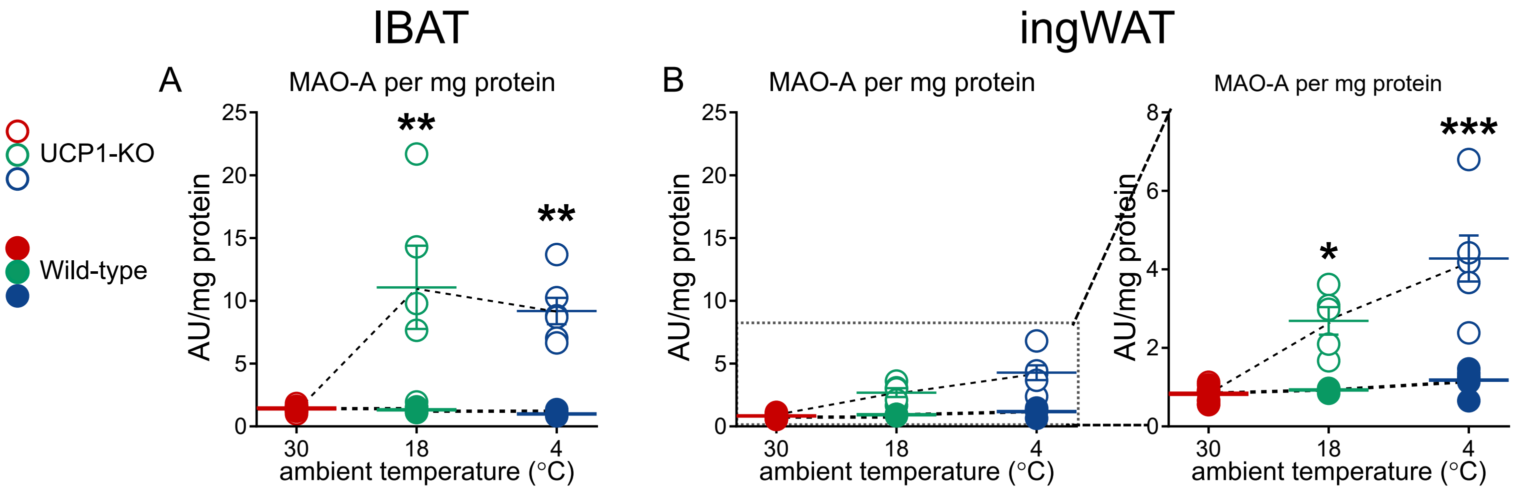

Figure S10. (A-B) Protein levels (quantification of immunoblots) of MAO-A in IBAT (A) and ingWAT (B) of the same animals as in Figures 1-8. The mean value in IBAT of wild-type mice acclimated to 4 °C was set to 1.0, and the levels in all other samples were expressed relative to this value. Each symbol represents a sample from one mouse. Values are means ± SEM. Where not visible, the error bars are smaller than the symbols. Full ANOVA statistics are given in Table S1. *Significant difference between wild-type and UCP1-KO mice for each tissue using two-way ANOVA followed by Tukey's multiple comparison test. *P < 0.05, **P < 0.01, ***P < 0.001. To facilitate comparisons of MAO-A levels between IBAT and ingWAT, the respective graphs were drawn with equal y-axis range. In the right panel of B, the graph was redrawn with optimal y-axis range.

Figure S11

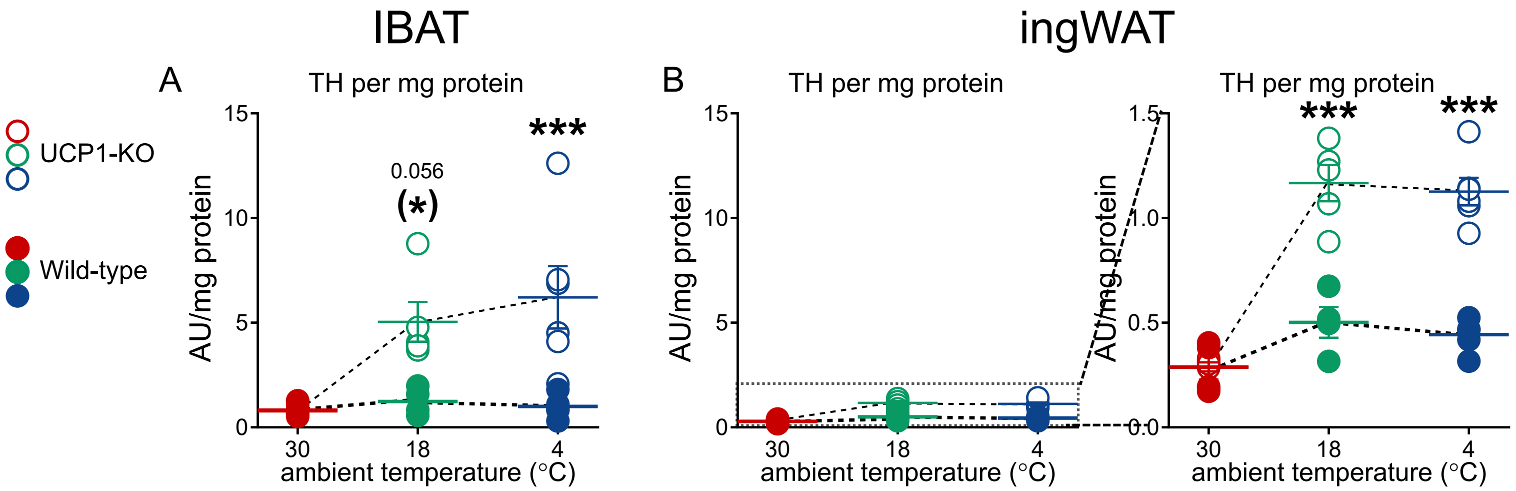

Figure S11. (A-B) Protein levels (quantification of immunoblots) of tyrosine hydroxylase (TH) in IBAT (A) and ingWAT (B) of the same animals as in Figures 1-8. The mean value in IBAT of wild-type mice acclimated to 4 °C was set to 1.0, and the levels in all other samples were expressed relative to this value. Each symbol represents a sample from one mouse. Values are means ± SEM. Where not visible, the error bars are smaller than the symbols. Full ANOVA statistics are given in Table S1. *Significant difference between wild-type and UCP1-KO mice for each tissue using two-way ANOVA followed by Tukey's multiple comparison test. ***P < 0.001. To facilitate comparisons of tyrosine hydroxylase levels between IBAT and ingWAT, the respective graphs were drawn with equal y-axis range. In the right panel of B, the graph was redrawn with optimal y-axis range.

Figure S12

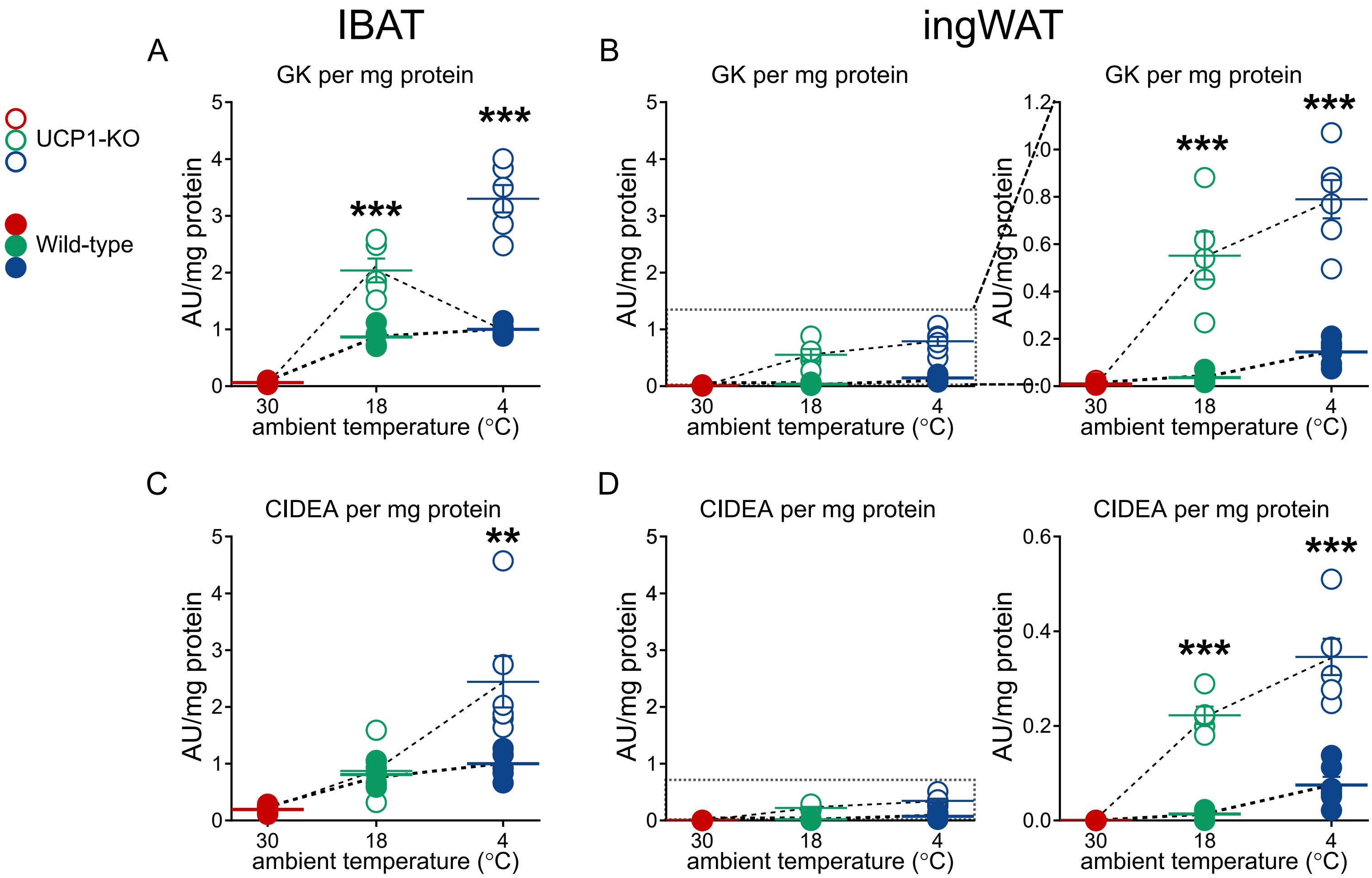

Figure S12. (A-D) Protein levels (quantification of immunoblots) of glycerol kinase (GK) in IBAT (A) and ingWAT (B) and CIDEA in IBAT (C) and ingWAT (D) of the same animals as in Figures 1-8. The mean value in IBAT of wild-type mice acclimated to 4 °C was set to 1.0, and the levels in all other samples were expressed relative to this value. Each symbol represents a sample from one mouse. Values are means ± SEM. Where not visible, the error bars are smaller than the symbols. Full ANOVA statistics are given in Table S1. *Significant difference between wild-type and UCP1-KO mice for each tissue using two-way ANOVA followed by Tukey's multiple comparison test. **P < 0.01, ***P < 0.001. To facilitate comparisons of examined proteins between IBAT and ingWAT, the respective graphs were drawn with equal y-axis range. In the right panels of B and D, the graphs were redrawn with optimal y-axis range.

Figure S13

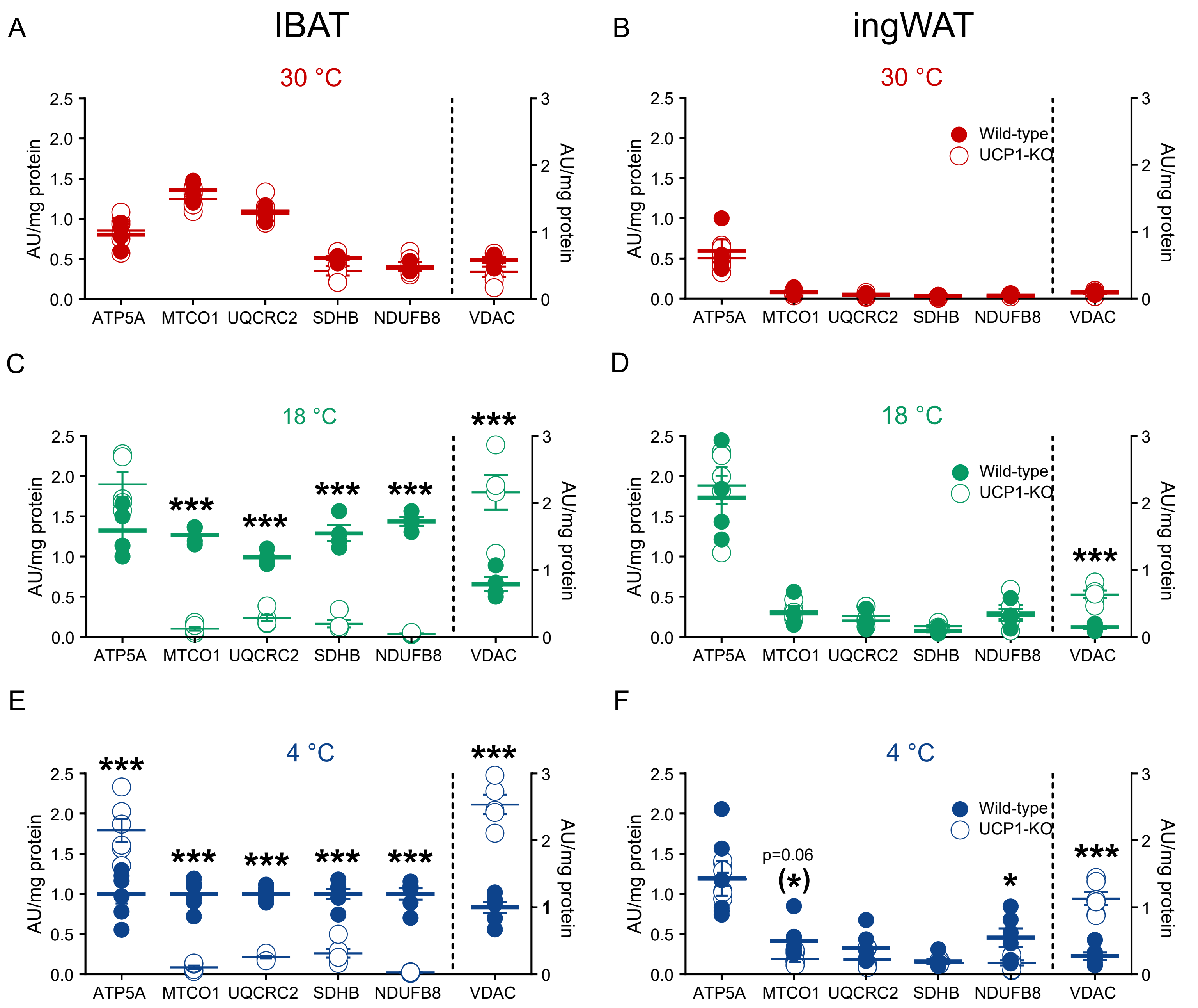

Figure S13. (A-F) The levels (quantification of immunoblots) of the indicated mitochondrial proteins in IBAT and ingWAT from animals acclimated to 30 °C (A and B), to 18 °C (C and D) and to 4 °C (E and F). The mean value for each protein in IBAT of wild-type mice acclimated to 4 °C was set to 1.0, and the levels in all other samples were expressed relative to this value. Each symbol represents a sample from one mouse. Values are means ± SEM. Where not visible, the error bars are smaller than the symbols. *Significant difference between wild-type and UCP1-KO mice for each tissue using two-way ANOVA followed by Tukey's multiple comparison test. *P < 0.05, ***P < 0.001. To facilitate comparisons of examined proteins between IBAT and ingWAT and also between the three temperatures, the respective graphs were drawn with equal y-axis range.
